# Supplementary material for: Low-dose aspirin to prevent preeclampsia and growth restriction in nulliparous women identified by uterine artery Doppler as at high risk of preeclampsia: A double blinded randomized placebo-controlled trial
Source: PLoS One. 2022 Oct 19;17(10):e0275129. doi: 10.1371/journal.pone.0275129 (PMC9581352; doi:10.1371/journal.pone.0275129)
Supplement: S1 Table — Results are numbers and percentages unless stated otherwise. (DOCX) [file pone.0275129.s001.docx]

S1 Table. Characteristics of the trial participants. Results are numbers and percentages unless stated otherwise.

|  | **Aspirin Group**  (N=550)  n (%) | **Placebo Group**  (n=550)  n (%) |
| --- | --- | --- |
|  |  |  |
| **Age**, years* | 28.3 ± 4.9 | 28.7 ± 4.7 |
| **Ethnicity** |  |  |
| White | 451 (82.0) | 453 (82.4) |
| Black | 81 (14.7) | 84 (15.3) |
| Other | 18 (3.3) | 13 (2.3) |
| **Body Mass Index*** | 22.2 [20.2 ; 25.0] | 22.0 [20.2 ; 25.0] |
| **Gestational age at randomization*** | 13.4 [12.8 ; 14.3] | 13.5 [12.8 ; 14.4] |
| **Lowest Pulsatility Index** | 2.01 [1.82 ; 2.27] | 2.02 [1.86 ; 2.27] |
| **Bilateral diastolic notching and lowest PI ≥ 1.7** | 110 (20.0) | 99 (18.0) |
| **Bilateral diastolic notching** | 29 (5.4) | 15 (2.7) |
| **Lowest PI ≥ 1.7** | 410 (74.6) | 436 (79.3) |
| **Medical history** |  |  |
| Diabetes mellitus type I | 1 (0.2) | 3 (0.5) |
| Diabetes mellitus II | 2 (0.4) | 0 (0.0) |
| Chronic hypertension | 3 (0.5) | 3 (0.5) |
| Autoimmune disease (other than lupus, or antiphospholipid syndrome) | 1 (0.2) | 3 (0.5) |
| **Gestational diabetes** | 15 (2.8) | 11 (2.0) |
| #Mean ± standard deviation (normally distributed variable)  *Median [Q25,Q75]  PI Pulsatility Index | | |
