# Supplementary material for: Low-dose aspirin to prevent preeclampsia and growth restriction in nulliparous women identified by uterine artery Doppler as at high risk of preeclampsia: A double blinded randomized placebo-controlled trial
Source: PLoS One. 2022 Oct 19;17(10):e0275129. doi: 10.1371/journal.pone.0275129 (PMC9581352; doi:10.1371/journal.pone.0275129)
Supplement: S2 Table — (DOCX) [file pone.0275129.s002.docx]

S2 Table. Outcomes according to randomization group

|  | Low-dose aspirin Group  (N=550)  n (%) | Placebo Group  (N=550)  n (%) | *P* | Difference of proportion and 95% CI | OR and 95% CI |
| --- | --- | --- | --- | --- | --- |
| Preeclampsia or birthweight ≤ 5th percentile |  |  |  |  |  |
| Analysis with imputation | 88 (16.0) | 79 (14.4) | 0.45 | 1.6 [-2.6 ; 5.9] | 1.14 [0.82 ; 1.58] |
| Complete-case analysis *(n=538 vs 543)* | 76 (14.1) | 72 (13.3) | 0.68 | 0.9 [-3.2 ; 5.0] | 1.08 [0.76 ; 1.52] |
| Preeclampsia *(n=541 vs 544)* | 28 (5.2) | 25 (4.6) | 0.66 | 0.6 [-2.0 ; 3.1] | 1.13 [0.65 ; 1.97] |
| Severe preeclampsia *(n=541 vs 544)* | 11 (2.0) | 11 (2.0) | 0.99 | 0.0 [-1.7 ; 1.7] | 1.01 [0.43 ; 2.34] |
| Preterm preeclampsia *(n=541 vs 544)* | 9 (1.7) | 12 (2.2) | 0.52 | -0.5 [-2.2 ; 1.1] | 0.75 [0.31 ; 1.79] |
| Preterm delivery before 34 weeks *(n=542 vs 544)* | 20 (3.7) | 17 (3.1) | 0.61 | 0.6 [-1.6 ; 2.7] | 1.19 [0.61 ; 2.29] |
| Anesthesia *(n=539 vs 537)* |  |  | 0.94 |  |  |
| None | 35 (6.5) | 33 (6.1) |  |  |  |
| Regional | 493 (91.5) | 494 (92.0) |  |  |  |
| General | 11 (2.0) | 10 (1.9) |  |  |  |
| Cesarean delivery *(n=542 vs 543)* | 123 (22.7) | 120 (22.1) | 0.81 | 0.6 [-4.4 ; 5.6] | 1.03 [0.78 ; 1.38] |
| Bleeding *(n=495 vs 500)* | 141 (28.5) | 121 (24.2) | 0.12 | 4.3 [-1.2 ; 9.8] | 1.25 [0.94 ; 1.66] |
| Epistaxis or gingival bleeding | 116 (82.3) | 94 (77.7) | 0.35 | 4.6 [-5.2 ; 14.3] | 1.33 [0.72 ; 2.45] |
| Metrorrhagia *(n=141 vs 121)* | 32 (22.7) | 39 (32.2) | 0.08 | -9.5 [-20.4 ; 1.3] | 0.62 [0.36 ; 1.07] |
| **Infant outcomes** |  |  |  |  |  |
| Birthweight ≤ 5th percentile (*n=540 vs 543*) | 58 (10.7) | 60 (11.1) | 0.87 | -0.3 [-4.0 ; 3.4] | 0.97 [0.66 ; 1.42] |
| Perinatal death *(n=542 vs 543)* | 8 (1.5) | 5 (0.9) | 0.40 | 0.6 [-0.7 ; 1.8] | 1.61 [0.52 ; 4.96] |
