# Supplementary material for: Low-dose aspirin to prevent preeclampsia and growth restriction in nulliparous women identified by uterine artery Doppler as at high risk of preeclampsia: A double blinded randomized placebo-controlled trial
Source: PLoS One. 2022 Oct 19;17(10):e0275129. doi: 10.1371/journal.pone.0275129 (PMC9581352; doi:10.1371/journal.pone.0275129)
Supplement: S3 Table — (DOCX) [file pone.0275129.s003.docx]

S3 Table: Serious adverse events

|  | Low-dose aspirin Group  (N=550)  n (%) | Placebo Group  (N=550)  n (%) | p |
| --- | --- | --- | --- |
| **Patients with at least one serious adverse event** | 112 (20%) | 120 (22%) | 0.55 |
|  |  |  |  |
| **Most frequent adverse events** |  |  |  |
| Threatened preterm labour | 19 (3.4%) | 23 (4.1%) |  |
| Maternal hypertensive complication | 14 (2.5%) | 19 (3.4%) |  |
| Maternal renal or urinary disorders | 3 (0.5%) | 3 (0.5%) |  |
| Metrorrhagia | 9 (7.8%) | 9 (7.8%) |  |
| Intra uterine growth restriction | 8 (1.4%) | 14 (2.5%) |  |
| Preterm delivery | 7 (1.3%) | 9 (1.6%) |  |
| Other neonatal disorder | 4 (0.7%) | 4 (0.7%) |  |
| Maternal death | 0 (0) | 0 (0) |  |
| Stillbirth or neonatal death | 10 (1.8%) | 5 (0.9%) |  |
