# Supplementary material for: Low-dose aspirin to prevent preeclampsia and growth restriction in nulliparous women identified by uterine artery Doppler as at high risk of preeclampsia: A double blinded randomized placebo-controlled trial
Source: PLoS One. 2022 Oct 19;17(10):e0275129. doi: 10.1371/journal.pone.0275129 (PMC9581352; doi:10.1371/journal.pone.0275129)

**PERASTUN**

**Prevention of pre-eclampsia**

**and of foetal growth retardation**

**with low-dose aspirin**

**in primiparous women with bilateral uterine artery notches**

**during the first trimester**

**Pragmatic randomised study**

**National PHRC 2008**

PHRN08 – FP – PERASTUN

**Version no. 3 of 27/11/2014**

**COORDINATING INVESTIGATOR**

Prof. Franck PERROTIN

Pôle de Gynécologie-Obstétrique,

Médecine Fœtale, Médecine et Biologie de la Reproduction

Centre Olympe de Gouges, CHRU de Tours

2, boulevard Tonnellé 37044 TOURS Cedex 9

Tel.: +33 (0)2.47.47.47.39 – Fax: +33 (0)2.47.47.38.01

Email: [franck.perrotin@med.univ-tours.fr](mailto:franck.perrotin@med.univ-tours.fr)

**SPONSOR**

Tours Regional University Hospital

Direction des Affaires Médicales et de la Recherche

Hôpital Bretonneau, CHRU de Tours

2, boulevard Tonnellé 37044 TOURS Cedex 9

**METHODOLOGY - QUALITY CONTROL**

# Centre for Clinical Investigation - INSERM 202

Hôpital Bretonneau, CHRU de Tours

2, boulevard Tonnellé 37044 TOURS Cedex 9

| SPONSOR | ***TOURS REGIONAL UNIVERSITY HOSPITAL*** |
| --- | --- |
| **CLINICAL TRIAL PROTOCOL**  ***PERASTUN*** | |
| TRIAL CODE | PHRN08 - FP / PERASTUN |
| EudraCT No. | 2011-003536-30 |
| FULL TITLE | Prevention of pre-eclampsia and of foetal growth retardation with low-dose aspirin in primiparous women with bilateral uterine artery notches during the first trimester.  Pragmatic randomised study. |
| PRODCIT/MOLECULE | KARDEGIC® 160 mg |
| PRINCIPAL INVESTIGATOR | Prof. Franck PERROTIN |
|  | Pôle de Gynécologie-Obstétrique, Médecine Fœtale, Médecine et Biologie de la Reproduction  Centre Olympe de Gouges - CHRU de Tours  2, Boulevard Tonnellé 37044 TOURS Cedex 9 |
| PROTOCOL VERSION No. | **3** |
| PROTOCOL DATE | **27/11/2014** |
| IEC | 2012-R8 |
| AFSSAPS | A120316-72 |
| CNIL | 912140 |

**Contents**

[*1.* *General information 10*](#__RefHeading___Toc99449606)

[1.1. Title 10](#__RefHeading___Toc99449607)

[1.2. Sponsor 10](#__RefHeading___Toc99449608)

[1.2.1. Identity 10](#__RefHeading___Toc99449609)

[1.2.2. Signing of the protocol on behalf of the sponsor 10](#__RefHeading___Toc99449610)

[1.2.3. Head of research for the sponsor 10](#__RefHeading___Toc99449611)

[1.3. Study coordination and monitoring 10](#__RefHeading___Toc99449612)

[1.4. Investigators 10](#__RefHeading___Toc99449613)

[1.4.1. Coordinating investigator 10](#__RefHeading___Toc99449614)

[1.4.2. Associate investigators 11](#__RefHeading___Toc99449615)

[1.5. Associate Scientists 13](#__RefHeading___Toc99449616)

[1.6. Coordinating pharmacy 13](#__RefHeading___Toc99449617)

[1.7. Pharmacovigilance 13](#__RefHeading___Toc99449618)

[1.8. Methodologist - biostatistician 13](#__RefHeading___Toc99449619)

[1.9. Supervisory committee 13](#__RefHeading___Toc99449620)

[2. Scientific rationale and general description of the research 15](#__RefHeading___Toc99449621)

[2.1. Pre-eclampsia 15](#__RefHeading___Toc99449622)

[2.2. Pathophysiology of placental insufficiency 15](#__RefHeading___Toc99449623)

[2.3. A disease of primiparous women 16](#__RefHeading___Toc99449624)

[2.4. Severe and early onset pre-eclampsia 16](#__RefHeading___Toc99449625)

[2.5. Uterine Doppler ultrasound screening 16](#__RefHeading___Toc99449626)

[2.5.1. Second trimester Doppler ultrasound 16](#__RefHeading___Toc99449627)

[2.5.2. First trimester Doppler ultrasound 17](#__RefHeading___Toc99449628)

[2.6. Primary prevention with aspirin 18](#__RefHeading___Toc99449629)

[2.6.1. General data 18](#__RefHeading___Toc99449630)

[2.6.2. Aspirin prevention trial in patients with a bilateral notch during the first trimester 18](#__RefHeading___Toc99449631)

[2.6.3. Effects according to the level of risk, dose and gestational age at initiation of treatment 18](#__RefHeading___Toc99449632)

[2.6.4. Side effects of aspirin 19](#__RefHeading___Toc99449633)

[2.6.5. Trials on primiparous women 19](#__RefHeading___Toc99449634)

[2.6.6. Subsequent research prospects 20](#__RefHeading___Toc99449635)

[2.7. Identifying the population at risk 20](#__RefHeading___Toc99449636)

[3. Summary of benefits, if any, and foreseeable and known risks to the persons involved in the research 21](#__RefHeading___Toc99449637)

[4. Effects 21](#__RefHeading___Toc99449638)

[5. Scientific literature and pertinent data will be used as a reference for the research 21](#__RefHeading___Toc99449639)

[6. Research objectives 25](#__RefHeading___Toc99449640)

[6.1. Primary objective 25](#__RefHeading___Toc99449641)

[6.2. Secondary objectives 25](#__RefHeading___Toc99449642)

[7. Research endpoints 25](#__RefHeading___Toc99449643)

[7.1. Primary endpoint 25](#__RefHeading___Toc99449644)

[7.1.1. Secondary endpoints 25](#__RefHeading___Toc99449645)

[8. Research design 26](#__RefHeading___Toc99449646)

[8.1. Description of the research methodology 26](#__RefHeading___Toc99449647)

[8.1.1. Experimental plan 26](#__RefHeading___Toc99449648)

[8.1.2. Description of measures taken to reduce and avoid bias 26](#__RefHeading___Toc99449649)

[8.1.3. Procedure under study 27](#__RefHeading___Toc99449650)

[8.1.4. Patient follow-up 27](#__RefHeading___Toc99449651)

[8.2. Expected duration of participation of persons and description of the timeline and duration of all test periods, including follow-up, where applicable 27](#__RefHeading___Toc99449652)

[9. Selection of research subjects 27](#__RefHeading___Toc99449653)

[9.1. Inclusion criteria 27](#__RefHeading___Toc99449654)

[9.2. Non-inclusion criteria 28](#__RefHeading___Toc99449655)

[9.3. Exclusion criteria 28](#__RefHeading___Toc99449656)

[9.4. Recruitment procedures 28](#__RefHeading___Toc99449657)

[9.4.1. Selection of recruiting sonographers 28](#__RefHeading___Toc99449658)

[9.4.2. Recruitment of patients 29](#__RefHeading___Toc99449659)

[10. Course the study 29](#__RefHeading___Toc99449660)

[10.1.1. Patient selection 29](#__RefHeading___Toc99449661)

[10.1.2. Inclusion visit 29](#__RefHeading___Toc99449662)

[10.1.3. Subsequent follow-up of pregnancy 30](#__RefHeading___Toc99449663)

[10.2. Rules for permanent or temporary discontinuation 30](#__RefHeading___Toc99449664)

[10.2.1. Discontinuation of participation 30](#__RefHeading___Toc99449665)

[10.2.2. Termination of part or all of the research 30](#__RefHeading___Toc99449666)

[10.2.3. Follow-up modalities for a patient leaving the study 30](#__RefHeading___Toc99449667)

[11. Treatments given to patients taking part in the research 31](#__RefHeading___Toc99449668)

[11.1. Description of the treatment(s) required to carry out the research 31](#__RefHeading___Toc99449669)

[11.1.1. Experimental medicinal product(s) 31](#__RefHeading___Toc99449670)

[11.2. Medicinal products and treatment authorised and prohibited under the protocol 32](#__RefHeading___Toc99449671)

[11.3. Method of monitoring compliance with treatment 32](#__RefHeading___Toc99449672)

[11.4. Storage conditions for investigational medicinal products 32](#__RefHeading___Toc99449673)

[11.4.1. Description of storage 32](#__RefHeading___Toc99449674)

[11.5. Stock management, replenishment 32](#__RefHeading___Toc99449675)

[12. Statistical considerations 32](#__RefHeading___Toc99449676)

[12.1. Number of patients to include 32](#__RefHeading___Toc99449677)

[12.2. Data analysis: general 34](#__RefHeading___Toc99449678)

[12.3. Description of samples at inclusion 35](#__RefHeading___Toc99449679)

[12.4. Primary endpoint analysis 35](#__RefHeading___Toc99449680)

[12.5. Secondary analyses 35](#__RefHeading___Toc99449681)

[12.6. Person responsible for the analysis 36](#__RefHeading___Toc99449682)

[13. Safety evaluation 36](#__RefHeading___Toc99449683)

[13.1. Procedures in place for recording and reporting adverse events 36](#__RefHeading___Toc99449684)

[13.1.1. Definitions 36](#__RefHeading___Toc99449685)

[13.1.2. Investigator's responsibility 37](#__RefHeading___Toc99449686)

[13.1.3. Sponsor's responsibility 39](#__RefHeading___Toc99449687)

[13.1.4. Supervisory Committee 41](#__RefHeading___Toc99449688)

[13.2. Modalities and duration of patient follow-up after the occurrence of adverse events 41](#__RefHeading___Toc99449689)

[14. Right of access to source data and documents 41](#__RefHeading___Toc99449690)

[14.1. Data access 41](#__RefHeading___Toc99449691)

[14.2. Source documents 41](#__RefHeading___Toc99449692)

[14.3. Data confidentiality 42](#__RefHeading___Toc99449693)

[15. Quality assurance and control 42](#__RefHeading___Toc99449694)

[16. Ethical considerations 43](#__RefHeading___Toc99449695)

[16.1. Independent Ethics Committee 43](#__RefHeading___Toc99449696)

[16.2. Substantive changes 43](#__RefHeading___Toc99449697)

[16.3. Patient information and written informed consent form 43](#__RefHeading___Toc99449698)

[16.4. Definition of the exclusion period 43](#__RefHeading___Toc99449699)

[16.5. Care related to the research 43](#__RefHeading___Toc99449700)

[16.6. Patient compensation 44](#__RefHeading___Toc99449701)

[16.7. Enrolment in the national biomedical research patient registry 44](#__RefHeading___Toc99449702)

[17. Data processing and storage of documents and data 44](#__RefHeading___Toc99449703)

[17.1. Case report form 44](#__RefHeading___Toc99449704)

[17.2. Data input and processing 44](#__RefHeading___Toc99449705)

[17.3. CNIL 44](#__RefHeading___Toc99449706)

[17.4. Archiving 44](#__RefHeading___Toc99449707)

[18. Financing and insurance 45](#__RefHeading___Toc99449708)

[18.1. Study budget 45](#__RefHeading___Toc99449709)

[18.2. Insurance 45](#__RefHeading___Toc99449710)

[19. Study feasibility 45](#__RefHeading___Toc99449711)

[20. Rules of publication 47](#__RefHeading___Toc99449712)

[21. Appendices 48](#__RefHeading___Toc99449713)

**Abstract**

| Title | Prevention of pre-eclampsia and of foetal growth retardation with low-dose aspirin in primiparous women with bilateral uterine artery notches during the first trimester. Pragmatic randomised study. |
| --- | --- |
| Rationale context | Pre-eclampsia is observed three to six times more frequently in primiparous women than in multiparous women. It is a serious condition for the mother (second cause of maternal mortality) and is a significant cause of morbidity. From a neonatal standpoint, it is a major cause of mortality and morbidity due to the prematurity it induces and the associated foetal growth retardation in more than one third of cases. In its recent report on "the effect of antiplatelet agents in preventing the onset of pre-eclampsia or its complications"; the Cochrane Collaboration concluded as follows: "Antiplatelet agents have moderate effects on the prevention of pre-eclampsia and its consequences. Further information is required to assess which women are most likely to benefit, when treatment is best started, and at what dose." (Duley *et al* Cochrane Database Syst Rev. 2007; (2):CD004659). |
| Primary Objective | To test the efficacy of low-dose aspirin (160 mg/day), initiated early on in pregnancy (≤ 15 WA completed), in primiparous patients determined to be "at risk" by the presence of a bilateral notch and/or a high uterine artery pulsatility index, in preventing the occurrence of pre-eclampsia during pregnancy or ≤5^th^ percentile neonatal hypotrophy. |
| Secondary Objectives | - To evaluate the effect of aspirin separately on each of the primary endpoint parameters (pre-eclampsia on the one hand and neonatal hypotrophy on the other hand)  - To evaluate the efficacy of aspirin in preventing early onset and severe forms of pre-eclampsia (occurring before 32 WA)  - To evaluate the efficacy of aspirin in preventing the need for birth before 34 WA, perinatal death (death between the 22^nd^ WA and postnatal day 7)  - To compare in each of the two groups (aspirin and placebo) the delivery route and the frequency of use of loco-regional analgesia  - To specify the maternal tolerance of the treatment  - To evaluate compliance with the treatment |
| Primary Endpoint | Occurrence of pre-eclampsia during pregnancy or birth weight ≤ 5^th^ percentile. |
| Secondary endpoints | - Gestational age at onset of early pre-eclampsia (onset before 32 WA) - Onset of severe pre-eclampsia - Induced prematurity (triggering and/or maturation, caesarean section) - Occurrence of perinatal death (death between the 22^nd^ WA and postnatal day 7) not attributable to a cause other than placental insufficiency - Delivery route: vaginal delivery or caesarean section. Elective caesarean sections (reason for planning) and emergency caesarean sections will be dissociated. - Use of loco-regional analgesia - Tolerance will be studied from the elements collected from adverse event reports, but also from the "patient" records submitted at the time of inclusion - Compliance will be evaluated by counting the treatments returned by patients |
| Methodology | Randomised, pragmatic, double-blind, parallel-group study |
| Inclusion criteria | - Women ≥ 18 years of age - Primiparous, i.e. never having had a pregnancy ≥ 22 WA - Having a singleton pregnancy - At a gestational age ≤ 15 WA completed i.e. 15^+6^ WA - Having a bilateral notch ≥ grade II and/or the lowest uterine artery pulsatility index value ≥ 1.7 during the first trimester ultrasound (between 45 and 84 mm CCL) - Having given their informed consent - Affiliates or beneficiaries of a social security scheme |
| Non-inclusion criteria | - Women wishing to terminate their pregnancy (abortion, with legal gestational age ≤ 14 WA) or for whom termination of pregnancy (therapeutic abortion or premature extraction) is envisaged before the gestational age of 37 WA - Having a foetal ultrasound abnormality detected during the first trimester ultrasound (foetal malformation or neck ≥ 95^th^ percentile) - On anticoagulant treatments - With known allergy or hypersensitivity to Kardegic® or any of its components - With primary or secondary haemostasis disease responsible for bleeding or at haemorrhagic risk - With active gastroduodenal ulcer - With lupus or anti-phospholipid syndrome |
| Processing Procedures | Patients in the experimental arm will receive 160 mg/day of Kardegic® from 11-15^+6^ WA and up to 34 WA. Patients in the control arm will receive a placebo that they will take according to the same dosage regimen as that defined for the experimental group. |
| Recruitment | Patients will be selected by ultrasound specialists after evaluation of their professional practices (EPP based on a tool implemented by CFEF [Collège Français d'Echographie Foetale - French college of foetal ultrasonography]) and sent to the investigators for inclusion in the study. |
| Exclusion period | There are no plans to exclude patients from participation in another clinical research other than a study on placental insufficiency. The exclusion period for studies of placental insufficiency ends within 72 hours of delivery. |
| Number of patients | 2,486 patients per arm, i.e. a total of 4,972 patients |
| Study duration | Duration of the inclusion period: **47 months**  Duration of participation for each patient: approximately 6 months  Total study duration: **53 months** |
| Expected benefits | To answer to the hypothesis of the efficacy of aspirin prescribed early (before the onset of trophoblast invasion) and at the highest dose proposed (in order to overcome a lack of efficacy by dose effect) in a "high-risk" subgroup of patients of 1/3 of primiparous women encompassing 75% of patients who will develop placental insufficiency. |

**List of abbreviations**

| AFSSAPS | Agence Française de Sécurité Sanitaire des Produits de Santé (French Health Products Safety Agency) |
| --- | --- |
| MA | Marketing Authorisation |
| CRA | Clinical Research Associate |
| GCP | Good Clinical Practice |
| CCTIRS | Comité Consultatif sur le Traitement de l'Information en matière de Recherche dans le domaine de la Santé (Advisory Committee on Information Processing in Material Research in the Field of Health) |
| CFEF | Collège Français d’Echographie Fœtale (French College of Foetal Ultrasonography) |
| IEC | Independent Ethics Committee |
| CNIL | Commission Nationale de l’Informatique et des Libertés (French National Commission for Information Technology and Civil Liberties). |
| CRF | Case Report Form |
| eCRF | Electronic Case Report Form |
| CRPV | Centre Régional de PharmacoVigilance (Regional PharmacoVigilance Centre) |
| ISC | Independent Supervisory Committee |
| AR | Adverse Reaction |
| SAE | Serious Adverse Event |
| SAR | Serious Adverse Reaction |
| SUSAR | Suspected Unexpected Serious Adverse Reaction |
| EPP | Evaluation of Professional Practices |
| RPH | Retroplacental Haematoma |
| HELPP | Haemolysis, Elevated Liver enzymes, Low Platelet count |
| CI | Confidence Interval |
| ICH | International Conference on Harmonization |
| SRN | State Registered Nurse |
| BMI | Body Mass Index |
| TA | Therapeutic Abortion |
| EAB | Elective Abortion |
| RI | Resistance Index |
| PI | Pulsatility Index |
| INSERM | Institut National de la Santé et de la Recherche Médicale (National Institute for Health and Medical Research) |
| CCL | Craniocaudal Length |
| IUFD | Intrauterine Foetal Death |
| RM | Reference Methodology |
| NK | Natural Killer |
| NNT | Number Needed to Treat |
| PlGF | Placental Growth Factor |
| PRF | Pulse Repetition Frequency |
| IHP | In-House Pharmacy |
| IUGR | Intrauterine Growth Retardation |
| SPC | Summary of Product Characteristics |
| RD | Risk Difference |
| RR | Relative Risk |
| WA | Weeks of Amenorrhoea |
| SUSAR | Suspected Unexpected Serious Adverse Reaction |
| CRT | Clinical Research Technician |
| VEGF | Vascular Endothelial Growth Factor |

# General information

## Title

"Prevention of pre-eclampsia and of foetal growth retardation with low-dose aspirin in primiparous women with bilateral uterine artery notches during the first trimester. Pragmatic randomised study."

## Sponsor

### Identity

**Tours Regional University Hospital**

37044 Tours cedex 9

### Signing of the protocol on behalf of the sponsor

**Ms Violaine MIZZI**

Director of Medical Affairs, Research and Quality

Hôpital Bretonneau, CHRU de Tours

### Head of research for the sponsor

**Ms Violaine MIZZI**

Director of Medical Affairs, Research and Quality

Hôpital Bretonneau, CHRU de Tours

## Study coordination and monitoring

# Centre for Clinical Investigation - INSERM 202

Hôpital Bretonneau, CHRU de Tours

2, boulevard Tonnellé 37044 TOURS Cedex 9

## Investigators

### Coordinating investigator

**Prof. Franck PERROTIN**

Pôle de Gynécologie-Obstétrique, Médecine Fœtale, Médecine et Biologie de la Reproduction

Centre Olympe de Gouges, CHRU de Tours

2, boulevard Tonnellé 37044 TOURS Cedex 9

### Associate investigators

**Dr Georges HADDAD**

Cabinet Mosaïque Santé

1, rue du Professeur Philippe Maupas 41260 La Chaussée St Victor

**Dr Jean-Pierre BERNARD**

Centre Européen de Diagnostic et d'Exploration de la Femme

15, rue Pottier 78150 Le Chesnay

**Prof. Yves VILLE**

**Centre de dépistage PRIMA FACIE**

Pôle Mère-Enfant Laennec

Hôpital Necker, AP-HP
149, rue de Sèvres 75015 PARIS

**Dr Guillaume BENOIST**

Service de Gynécologie-Obstétrique et Médecine de la Reproduction

Hôpital Côte de Nacre, CHU de Caen
Avenue de la Côte de Nacre 14033 Caen Cedex 9

**Dr Muriel DORET**

Service de Gynécologie-Obstétrique

Hôpital Femme, Mère, Enfant - Groupement Hospitaliers Est, Hospices Civils de Lyon

59, bd Pinel 69677 Bron

**Dr Pascal MEGIER**

Service de Gynécologie-Obstétrique

Hôpital Porte Madeleine, CHR d’Orléans

1, rue Porte Madeleine 45032 Orléans Cedex 1

**Prof. Didier LEMERY**

Pôle Gynécologie, Obstétrique, Reproduction Humaine

Hôpital d’Estaing, CHU de Clermont-Ferrand

1, place Lucie-Aubrac 63003 Clermont-Ferrand Cedex 1

**Prof. Pierre MARES**

Service de Gynécologie-Obstétrique

Hôpital Carémeau, CHRU de Nîmes

**Place du Pr Robert Debré 30029 Nîmes cedex 9**

**Dr Anaig FLANDRIN**

Département de Gynécologie-Obstétrique

Hôpital Arnaud de Villeneuve, CHRU de Montpellier

371, avenue du Doyen Gaston Giraud 34295 Montpellier Cedex 5

**Prof. Christophe VAYSSIERE**

Pôle Femme Mère Couple

Hôpital Paule de Viguier, CHU de Toulouse

330, avenue de Grande Bretagne 31059 Toulouse cedex 9

**Prof. Dominique DALLAY**

Service de Gynécologie-Obstétrique et Reproduction

Groupe Hospitalier Pellegrin, CHRU de Bordeaux

Place Amélie Raba-Léon 33000 Bordeaux

**Prof. Véronique HOUFFLIN-DEBARGE**

Pôle Femme, Mère et nouveau-né

Hôpital Jeanne de Flandre, CHRU de Lille

Avenue Eugène Avinée 59037 Lille

**Dr Norbert WINER**

Service de gynécologie-obstétrique

Hôpital Mère-Enfant, CHRU de Nantes
38, boulevard Jean-Monnet 44093 Nantes Cedex 1

**Dr Véronique EQUY**

Département de Gynécologie-Obstétrique

Hôpital Couple Enfant, CHRU de Grenoble

CS10217 - 38043 Grenoble Cedex 9

**Dr Marie-Thérèse CHEVE**

Service de Gynécologie-Obstétrique

CH du Mans

194, avenue Rubillard 72037 Le Mans Cedex 9

**Dr Edwin QUARELLO**

Pôle Parents Enfants Sainte Monique

Hôpital Saint Joseph

26, boulevard de Louvain 13285 Marseille Cedex 8

**Dr Luc DURIN**

Service de Gynécologie-Obstétrique

Polyclinique du Parc

20, avenue Capitaine Georges Guynemer 14052 Caen Cedex 4

**Dr Anne PAUMIER**

Service de Gynécologie-Obstétrique

Polyclinique de l’Atlantique

Avenue Claude Bernard 44819 Saint Herblain Cedex

**Dr Bruno SCHAUB**

Maison de la Femme, de la Mère et de l'Enfant

CHU de **Fort-de-France**

**CS 90632 - 97261 Fort-de-France Cedex -** Martinique

## Associate Scientists

**Dr. Nicolas FRIES**

President of the French College of Foetal Ultrasonography (CFEF)

Cabinet Les Tonnelles

131, avenue de Lodève 34080 **Montpellier**

## Coordinating pharmacy

**Internal Pharmacy (IP)**

Hôpital Bretonneau, CHRU de Tours

2, boulevard Tonnellé 37044 TOURS Cedex 9

## Pharmacovigilance

**Dr Annie-Pierre JONVILLE-BERA**

Service de Pharmacologie Clinique

Hôpital Bretonneau, CHRU de Tours

2, boulevard Tonnellé 37044 TOURS Cedex 9

## Methodologist - biostatistician

# Dr Bruno GIRAUDEAU

# Centre for Clinical Investigation - INSERM 202

Hôpital Bretonneau, CHRU de Tours

2, boulevard Tonnellé 37044 TOURS Cedex 9

## Supervisory committee

An independent supervisory committee, comprised of 3 non-investigator experts, will be formed. It will consist of two obstetricians independent of the study and one methodologist:

**Prof. Alexandra BENACHI**

Service de Gynécologie-Obstétrique et Médecine de la Reproduction

Hôpital Antoine Béclère, APHP

157, rue de la Porte de Trivaux 92141 CLAMART Cedex

**Prof. Patrick ROZENBERG**

Service de Gynécologie-Obstétrique

CHI Poissy/Saint Germain-en-Laye, APHP

10, rue du Champ Gaillard 78303 Poissy Cedex

**Véronique RIVAIN**

Biomathematics - Biostatistics Laboratory

UFR des Sciences Pharmaceutiques, Université de Nantes
1, rue Gaston Veil 44035 Nantes Cedex 1

# Scientific rationale and general description of the research

Pre-eclampsia and intrauterine growth retardation (IUGR) represent maternal and foetal translations of a placental disease often referred to as global placental insufficiency.

## Pre-eclampsia

Pre-eclampsia, characterised by the onset of high blood pressure with proteinuria after 20 weeks of amenorrhoea (WA), in a previously normotensive pregnant woman affects about 1 to 3% of multiparous and 5 to 7% of primiparous women [1-3]. This is a serious condition, due to its maternal and foetal complications. Indeed, it is considered to be responsible for approximately 10-15% of maternal deaths in industrialised countries. In France, pre-eclampsia is the second leading cause of maternal mortality after postpartum haemorrhage [4]. The severity of pre-eclampsia is also due to the severity of its maternal complications causing significant morbidity (eclampsia, retro-placental haematoma (RPH), pulmonary oedema, renal failure, etc.).

Its consequences are also significant at the neonatal level, because of the IUGR associated with it in more than one third of cases, but also because of the prematurity induced by the need to manage maternal complications, the only treatment of which is often to terminate the pregnancy. Mortality and perinatal morbidity are 5 to 30 times higher than for children born to non-pre-eclamptic mothers and whose birth weight is normal (between the 10^th^ and 90^th^ percentiles) [5,6].

## Pathophysiology of placental insufficiency

Although the precise pathophysiology of placental insufficiency has not yet been fully elucidated, it is recognized that it results from an insufficiency of uteroplacental perfusion secondary to a defect in trophoblast invasion that usually occurs between 16 and 20 WA [7,8]. This trophoblast invasion corresponds to the penetration of the lumen of maternal spiral arteries by extravillous trophoblast cell cords, originating from the base of the placental anchoring villi. This invasion leads to partial destruction of the wall of the spiral arteries, which then stretch to increase placental flow by reducing the vascular resistance.

In the absence of such adaptation, uteroplacental vascularisation remains highly resistant and the resulting placental ischemia causes a reduction in foetal-maternal exchanges and a release, into the maternal circulation, of various substances causing disruptions in endothelial function and maternal clinical presentation of pre-eclampsia [9,10]. Activation of platelets and the coagulation system may occur early in the onset of placental insufficiency, before clinical symptoms develop [11,12]. Deficient intravascular production of prostacyclin, a vasodilator and antiplatelet agent, along with excess production of thromboxane A2, a vasoconstrictor and platelet aggregation stimulator, could also be demonstrated [13,14]. More recently, significant variations in certain pro-angiogenic growth factors such as the Placental Growth Factor (PlGF), Vascular Endothelial Growth Factor (VEGF) and anti-angiogenic factors such as soluble VEGF receptor (sFLT-1), have been demonstrated and their involvement has been highlighted from the early stages of the disease [15-17]. The observation of these phenomena, and more specifically those involving platelet aggregation, led to the hypothesis that antiplatelet agents, in particular aspirin used at low doses, could prevent or delay the occurrence of placental insufficiency.

## A disease of primiparous women

Pre-eclampsia is observed three to six times more frequently in primiparous women than in multiparous women. This observation makes the primiparous population, *per se*, a high-risk population [18, 19]. Moreover, the absence (by definition) of any reference obstetric history in this group deprives the clinician of some of the elements enabling them to modulate the risks in relation to the patient's clinical history.

Several hypotheses have been posited to explain this higher risk in primiparous patients [7]. The first hypothesis is that of insufficient adaptation of the spiral arteries after trophoblast invasion during the first pregnancy. This explanation could account for the fact that only 15% of placental insufficiencies recur during a subsequent pregnancy, the adaptation being more complete during subsequent pregnancies, the spiral arteries having already undergone initial remodelling of their vascular wall. The second explanation involves a partially inadequate maternal immune response to trophoblast cells. Indeed, during trophoblast invasion, the extravillous cytotrophoblast cells will express some of the paternal antigens to the maternal immunocompetent cells (NK lymphocytes). Modulation of the lymphocyte response involves a "tolerance" to paternal antigens that appears to develop in the maternal organism in connection with repeated exposure to the same antigens expressed on the surface of spermatozoa. This explanation is supported by several large-scale epidemiological studies that have demonstrated a link between the frequency of pre-eclampsia in primiparous patients and the age of the first unprotected sexual intercourse with the procreator of the current pregnancy [20].

## Severe and early onset pre-eclampsia

Due to the more severe maternal and neonatal consequences (induced prematurity, morbidity, mortality), two progressive entities of pre-eclampsia are currently increasingly dissociated; severe and early onset pre-eclampsia, characterised by onset before 32 WA and at least one of the usual criteria of severity (associated IUGR, systolic blood pressure ≥ 160 mmHg and/or diastolic ≥ 110 mmHg, proteinuria ≥ 5 g/day, diuresis ≤ 400 mL/day or ≤ 20 mL/h, HELLP syndrome, eclampsia, acute pulmonary oedema, RPH, intrauterine foetal death (IUFD)) and later onset pre-eclampsia, whose maternal picture may, however, in some cases be severe [4].

## Uterine Doppler ultrasound screening

### Second trimester Doppler ultrasound

The lack of adaptation of the spiral arteries under the effect of trophoblast invasion results in the persistence of high-resistance uteroplacental circulation. In the uterine arteries, this translates to high resistance or pulsatility indices [21,22]. The existence of a notch (protodiastolic notch on the Doppler spectrum) is also attributed to this insufficient adaptation of the spiral arteries. Studied by many teams, uterine Doppler ultrasonography has thus demonstrated its effectiveness in screening for pre-eclampsia with a more or less strong sensitivity and specificity depending on the populations studied, the thresholds retained for the uterine resistance indices and the definition of the condition under exploration [23]. In a multi-centre study of 8,335 singleton pregnancies at 23 WA, the sensitivity of a pulsatility index ≥ 95^th^ percentile (approximately 5% false positives) when screening patients who develop pre-eclampsia was 40.7% (90.0% when screening for early onset and severe forms of pre-eclampsia) [23]. Screening during the second trimester, however, does not open the possibility of management other than increased surveillance, the effectiveness of treatments such as aspirin being questionable [24].

### First trimester Doppler ultrasound

For this reason, many teams turned to first trimester screening [25-27]. This development goes hand in hand with the development between 11 and 14 WA of screening policies in other areas such as that of Down syndrome, for example (measurement of foetal neck).

Unlike during the second trimester, changes in uteroplacental circulation between 11 and 14 WA are still only very partial, the invasion of the spiral arteries by trophoblast cells being incomplete or even only beginning. It is therefore understandable that the performance of the Doppler ultrasound of the uterine arteries is weaker. Thus, in a large study involving 3,324 non-selected patients, the value of a pulsatility index (PI) ≥ 95^th^ percentile gives only a sensitivity not exceeding 27% for the screening of pre-eclampsia (60% for early onset and severe pre-eclampsia requiring extraction before 32 WA) and 11.7% for the screening of IUGR (27.8% for IUGR requiring extraction before 32 WA) [26]. If a lower specificity value is allowed (10% false positives), sensitivity increases, in this same population, to 52% [22].

Recently, several publications have pointed out that, instead of focusing on the average PI of the two uterine arteries, which can be influenced by the lateralisation of placental insertion, it is the lowest PI on the side where the placenta is implanted that has the highest sensitivity value, mainly for early pre-eclampsia [28-30]. It is relatively difficult to give an upper-bounding threshold of this lowest PI between right and left uterine arteries. However, in a study of more than 9,000 low-risk singleton pregnancies, with a pre-eclampsia prevalence of 2%, the median (interquartile range) of the lowest PI was 1.68 (1.24-2.16) for patients who developed pre-eclampsia versus 1.4 (1.14-1.72) for patients who were not affected [30]. For patients developing early onset pre-eclampsia, this value was 2.26 (1.82-2.41). Based on this broad study, a low PI threshold ≥ 1.7 seems to have a good discriminating value since it concerns less than 25% of patients who will not develop pre-eclampsia and more than 50% of those who will progress to PE (more than 75% of those who will have early onset pre-eclampsia).

According to studies, the presence of bilateral notch between 11 and 14 WA is reported in 30 to 55% of patients. The sensitivity of the bilateral notch of the uterine arteries for the screening of pre-eclampsia varies between 45 and 75% [25]. Several points may explain this wide variation in notch performance. First, the "subjective" nature of the identification of the protodiastolic notch: some authors consider that a notch is present only when it is visualized over several consecutive cycles (up to 5 for some) [27]. The more or less pronounced nature of the protodiastolic notch on the spectrum may also explain this variation. Finally, it also appears that the frequency with which such a notch is found decreases with the gestational age since, in the study by O. Gomez *et al*., it changed from 48% at 11 WA to 28% at 14 WA [27].

In a study conducted at our centre on a population of 435 primiparous patients between 11 and 14 WA, a grade II and III bilateral notch was observed in 35% of patients and presented a sensitivity of 70% for the screening of pre-eclampsia and of 50% for the screening of IUGR [31].

It is thus clear that the Doppler ultrasound of the uterine arteries during the first trimester, while it is an imperfect tool in the low-risk population, may serve, in a high or intermediate risk population, to select a subgroup of patients for whom the high prevalence of pre-eclampsia or IUGR is such that it may justify preventive treatment if the benefit-risk ratio is favourable.

## Primary prevention with aspirin

### General data

Numerous treatments have been tested for the prevention of placental insufficiency. Thus, vitamins C and E, along with calcium, have been proposed, though their effects were found to be disappointing [32-34]. To date, only low-dose aspirin (between 50 and 160 mg/day) has demonstrated its - limited- efficacy in the prevention of pre-eclampsia, or of the perinatal consequences of placental insufficiency [35]. Early studies involved overly small populations, thus partially explaining the importance of the effect ascribed to aspirin [36]. The high degree of variability in the efficacy of aspirin in published studies, is primarily attributed to the pronounced heterogeneity of study populations, and thus of the incidence of the disease, but also to wide variations in the gestational age at which aspirin was initiated and in the dose prescribed [37].

The effect attributed to aspirin in the meta-analyses of previously published studies is a 10 to 17% decrease in the incidence of pre-eclampsia, an 8 to 10% decrease in the risk of significantly premature birth (less than 34 WA) and a 10 to 14% decrease in the risk of severe perinatal event (IUFD, neonatal death). Thus, in the meta-analysis of L.M. Askie et al. regarding the data of 32217 patients, taken from 31 studies of the primary prevention of pre-eclampsia, the relative risk (RR) of developing pre-eclampsia is of 0,90 (95% CI: 0.85-0.96), the RR of giving birth before 34 WA is of 0.90 (0.83-0.98) and the RR of occurrence of an unfavourable perinatal outcome is of 0.90 (0.85-0.96) [38].

The conclusions of the systematic review of the Cochrane Library, encompassing 59 publications (37,560 patients) are a 17% reduction in the risk of pre-eclampsia (46 trials, 32,891 patients, RR 0.83, 95% CI 0.77-0.89), with a number of patients requiring treatment to avoid pre-eclampsia (NNT) estimated at 72 patients (52, 119). This compilation of randomised trials also noted an 8% reduction in the risk of premature birth (29 trials, 31,151 patients, RR 0.92, 95% CI: 0.88-0.97); NNT 72 (52, 119), a 14% reduction in perinatal intrauterine foetal death (40 trials, 33,098 patients, RR 0.86, 95% CI: 0.76-0.98); NNT 243 (131, 1,666) and a 10% reduction in hypotropohic neonates (36 trials, 23,638 patients, RR 0.90, 95% CI: 0.83-0.98) [35].

### Aspirin prevention trial in patients with a bilateral notch during the first trimester

Only one trial, addressing only very imperfectly the question, has been published, due primarily to a lack of power related to population size. In this controlled randomised study, the prescription of a 0.5 mg/kg dose of aspirin in patients with bilateral uterine notching between 11 and 14 WA (30% of primiparous women) was accompanied by an 80% reduction in the risk of pre-eclampsia (RR 0.2; 95% CI: 0.13-0.78) with no significant reduction in the risk of IUGR [39].

### Effects according to the level of risk, dose and gestational age at initiation of treatment

Part of the controversy surrounding the efficacy of aspirin is due to the pronounced heterogeneity of patients in the published studies, to the variability of doses at which aspirin is used and to the variability of terms at initiation of treatment. Thus, doubts persist concerning the enhanced efficacy of aspirin used at higher doses (100 to 150 mg rather than 50 or 75 mg), initiated at an earlier gestational age (before 16 WA rather than after), or used in patients at higher risk [38].

In the systematic Cochrane analysis, there are no statistically significant differences in the RR of pre-eclampsia according to the maternal risk level. However, a significant increase in the reduction of the absolute risk of pre-eclampsia in high-risk patients is observed (risk difference (RD) -5.2% (-7.5, -2.9), NNT 19 (13, 34)) in comparison to lower risk patients (RD -0.84 (-1.37, -0.3), NNT 119 (73, 333)) [35].

In the meta-analysis of the individual data of patients included in one of the 31 prospective randomised trials for which these data were available (32,217 patients), LM Askie et al. concluded that no single patient subgroup had a higher probability of deriving a positive effect from aspirin prescription. As such, it does not appear certain that aspirin should be reserved for the most at-risk patients [35]. Similarly, in this analysis, no significant differences in the effect of aspirin were observed depending on whether the treatment was initiated before 20 WA or after 20 WA and on whether the dose was ≤75 mg/day or >75 mg/day, even though in both cases, there was a trend towards a greater effect with earlier treatments and higher doses.

In order to ensure maximum efficacy while remaining within the known maternal and foetal safety zone, we opted to use aspirin at a dose of 160 mg/day and to initiate treatment at a gestational age ≤15 completed WA.

### Side effects of aspirin

In the Cochrane Library review (12 studies, 22,309 patients), no maternal or foetal accidents were reported [32]. In particular, no increases in the frequency of retro-placental haematoma or maternal haemorrhage were observed. Some trials, however, reported an increased frequency of maternal bleeding. This was the case of the ERASME study, in which the frequency of minor bleeding was significantly (though moderately) higher (RR 1.25, 95% CI: 1.03-1.54) [24].

With respect to the foetus, no increased risk of bleeding, particularly intra-cranial bleeding, was highlighted in the meta-analyses of published trials. In light of the results of the exposure registers of numerous pregnancies to aspirin, including during embryogenesis, before 11 WA, there are no formal arguments in favour of an increased risk of malformation.

In our trial, maternal haemorrhagic events will be collected.

### Trials on primiparous women

Only one prospective randomised trial has tested, on 3,294 primiparous women, the efficacy of systematic prevention of pre-eclampsia by treatment with 100 mg aspirin. The incidence of pre-eclampsia was not reduced in the aspirin group (RR 1.08, 95% CI: 0.64-1.83). Similarly, no significant reduction in the frequency of arterial hypertension (all forms), of maternal pre-eclampsia complications, in the incidence of perinatal death or in birth weights lower than the 10^th^ percentile was highlighted [23,40]. In this study, the prevalence of pre-eclampsia was only of 1.6% in the control group and patients were included between 14 and 20^+6^ WA (mean gestational age 16^+3^ WA).

In this same trial, a third randomisation arm (Doppler ultrasound at 22-24 WA and aspirin in the event of bilateral notching or mean resistance index greater than 0.61) was offered to a fraction of the patients due to the availability of uterine Doppler ultrasonography in only half of the investigation centres. By comparison with the placebo group, this study showed that such a strategy applied to primiparous women did not entail a significant reduction in the risk of pre-eclampsia (RR 1.55, 95% CI: 0.7-3.3), of hypotrophy ≤3^rd^ percentile or of perinatal death [40].

### Subsequent research prospects

Despite more than 30 years of prospective randomised trials and the involvement of more than 30,000 patients in these trials, the controversy surrounding the efficacy of aspirin in the prevention of pre-eclampsia and IUGR persists [37]. In its recent report on "the effect of antiplatelet agents in preventing the onset of pre-eclampsia or its complications"; the Cochrane Collaboration concluded as follows: "Antiplatelet agents have moderate effects on the prevention of pre-eclampsia and its consequences. Further trials are required to assess which patients are most likely to benefit, when treatment is best started, and at what dose." [35].

Recent advances in the early screening of pre-eclampsia, using uterine artery Doppler ultrasonography during the first trimester, open a new line of research into the efficacy of aspirin in primary prevention. The theoretical benefits are two-fold:

- This is early screening and should thus enable the initiation of treatment at a gestational age when trophoblast invasion has not yet begun. It will thus have a greater chance of being more effective.
- It does not add the need for additional examinations relative to the usual follow-up of pregnancy, only an evaluation involving a simple examination (Doppler ultrasound of the uterine arteries) that falls within the expertise of most professionals performing obstetrical ultrasonography.

## Identifying the population at risk

Due to the good tolerance of low-dose aspirin and to the severity of the maternal and foetal consequences of pre-eclampsia, we considered it essential to choose a test selecting the patients to treat with high sensitivity, despite a poor specificity.

Moreover, to facilitate patient selection, we selected a number of easily identifiable Doppler criteria (notch for which the lowest PI ≥1.7).

We also deliberately opted to ignore screening strategies combining Doppler ultrasonography and medical history or Doppler ultrasonography and biochemical assays (PAPP-A, free β-hCG, endothelial growth factors). For the former, the reasons were, besides the complexity of some of the selected criteria, the fact that primiparous women, by definition, have no obstetrical history (that with the greatest weight) and that the other factors (obesity, chronic hypertension, diabetes, Afro-American ethnicity) pertain only to a small proportion of patients. As for the strategies based on a combination of Doppler ultrasound of the uterine arteries with biochemical marker assays, their evaluation is currently incomplete and their implementation on a large population is too expensive to allow their use in clinical practice [41-42].

In our starting hypothesis, we thus selected our study population considering that the presence of bilateral notching on the Doppler ultrasound of the uterine arteries and/or the lowest PI ≥1.7 during the first trimester, would allow us to detect 75% of pre-eclamptic patients or those with intrauterine growth retardation with a specificity of 65% [31].

# Summary of benefits, if any, and foreseeable and known risks to the persons involved in the research

The expected benefits of preventive treatment are a 20% reduction in the incidence of pre-eclampsia and hypotrophy (≤5^th^ percentile). We also hope for a reduction in the frequency of severe pre-eclampsia and its maternal complications, premature births and perinatal deaths.

The risks are dominated by a slightly increased risk of minimal maternal bleeding (epistaxis, gingivorrhagia, vaginal bleeding, rectal bleeding, bruising). There are no reports in the literature of a significant increase in major maternal bleeding (RPH, postpartum haemorrhage, etc.) or of a significant increase in major or minor neonatal bleeding. Exposure of a large number of pregnancies to low-dose aspirin eliminates the risk of foetal malformations when used at ≤ 150 mg/day [35]. Moreover, initiation of treatment in our trial after the ultrasound of 11-14 WA limits exposure during embryogenesis.

Patients will be clearly informed that due to the antiplatelet effect and an increased risk of spinal haematoma in such cases, they may not be able to benefit from locoregional (epidural) anaesthesia if the discontinuation of treatment is ≤ 8 days at the time of use (especially in case of premature delivery).

# Effects

To answer to the hypothesis of the efficacy of aspirin prescribed early (before the onset of trophoblast invasion) and at the highest dose proposed (in order to overcome a lack of efficacy by dose effect) in a "high-risk" subgroup of patients of 1/3 of primiparous women encompassing 75% of patients who will develop placental insufficiency.

# Scientific literature and pertinent data will be used as a reference for the research

1. World Health Organization International Collaborative Study of Hypertensive Disorders of Pregnancy. Geographic variations in the incidence of hypertension in pregnancy. Am J Obstet Gynecol 1988; 158: 80-83
2. World Health Organization. Make every mother and child count. World Health Report 2005. WHO: Geneva, 2005.
3. ACOG Committee on Practice Bulletins – Obstetrics. ACOG practice Bulletin: diagnosis and management of pre-eclampsia and eclampsia: number 33, January 2002, Obstet Gynecol 2002; 99: 159-167.
4. Philibert M, Boisbras F, Bouvier-Colle MH. Epidémiologie de la mortalité maternelle en France, de 1996 à 2002 : fréquence, facteurs et causes. La mortalité maternelle en France: bilan et perspectives - Numéro thématique - BEH thématique 2006; 50: 392-295
5. Mari G, Hanif F. Intrauterine growth restriction: how to manage and when to deliver. Clin Obstet Gynecol 2007; 50: 497-509.
6. Alberry M, Soothill P. Management of fetal growth restriction. Arch Dis Child Fetal Neonatal Ed. 2007; 92: F62-67.
7. Redman CW.Current topic: pre-eclampsia and the placenta. Placenta 1991;12: 301-308.
8. Redman CW. Immunology of preeclampsia. Semin Perinatol. 1991; 15: 257-62.
9. Roberts JM, Cooper DW. Pathogenesis and genetics of pre-eclampsia. Lancet 2001; 357: 536.
10. Bartha JL, Romero-Carmona R, Escobar-Lompart M, Comino-Delgado R. The relationships between leptin and inflammatory cytokines in women with pre-eclampsia. BJOG 2001; 108: 1272-6.
11. Redman CW, Bonnar J, Beilin L. Eraly platelet consumption in pre-eclampsia. Br Med J 1978; i: 467-9.
12. Janes SL, Kyle PM, Redman C, Goodall AH. Flow cytometric detection of activated platelets in pregnant women prior to the development of pre-eclampsia. Thromb Haemost 1995; 74: 1059-63.
13. Bussolino F, Benedetto C, Masobrio M, Camussi G. Maternal vascular prostacyclin activity in pre-eclampsia. Lancet 1980; 2: 702.
14. Masotti G, Galanti G, Poggesi L, Abbate R, Neri Serneri GG. Differential inhibition of prostacyclin production and platelet aggregation by aspirin. Lancet 1979; 2: 1213
15. Luttun A, Carmeliet P. Soluble VEGF receptor flt-1: the elusive preeclampsia factor discovered. J Clin Invest 2003; 111: 600-2
16. Bujold E, Romero R, Chaiworapongsa T. Evidence supporting that the excess of the SVEGFR-1 concentration in maternal plasma in preeclampsia has an uterine origin. The J. Maternal and fetal and neonat Med 2005; 18 : 9-16.
17. Krysiak O, Bretschneider A, Zong E, Webb J, Soluble Vascular Endothelial Growth Factor-1 (sFLT-1) Mediates downregulation of FLT-1 and prevents activated neutrophils from women with preeclampsia from additional migration by VEGF. Circ Res 2005 Nov 3.
18. NHBPE. National High Blood Pressure Education Program Working Group on high blood pressure in pregnancy. Am J Obstet Gynecol 1990; 163: 1691-1712.
19. Odegard, R. A.; Vatten, L. J.; Nilsen, S. T.; Salvesen, K. A., and Austgulen, R. Risk factors and clinical manifestations of pre-eclampsia. BJOG. 2000 Nov; 107(11):1410-6
20. Einarsson, J. I.; Sangi-Haghpeykar, H., and Gardner, M. O. Sperm exposure and development of preeclampsia. Am J Obstet Gynecol. 2003 May; 188(5):1241-3.
21. Campbell, S.; Diaz-Recasens, J.; Griffin, D. R.; Cohen-Overbeek, T. E.; Pearce, J. M.; Willson, K., and Teague, M. J. New doppler technique for assessing uteroplacental blood flow. Lancet. 1983 Mar 26; 1(8326 Pt 1):675-7.
22. Yu, C. K.; Smith, G. C.; Papageorghiou, A. T.; Cacho, A. M., and Nicolaides, K. H. An integrated model for the prediction of preeclampsia using maternal factors and uterine artery Doppler velocimetry in unselected low-risk women. Am J Obstet Gynecol. 2005 Aug; 193(2):429-36.
23. Papageorghiou, A. T.; Yu, C. K.; Erasmus, I. E.; Cuckle, H. S., and Nicolaides, K. H. Assessment of risk for the development of pre-eclampsia by maternal characteristics and uterine artery Doppler. BJOG. 2005 Jun; 112(6):703-9.
24. Subtil, D.; Goeusse, P.; Puech, F.; Lequien, P.; Biausque, S.; Breart, G.; Uzan, S.; Marquis, P.; Parmentier, D., and Churlet, A. Aspirin (100 mg) used for prevention of pre-eclampsia in nulliparous women: the Essai Regional Aspirine Mere-Enfant study (Part 1). BJOG. 2003 May; 110(5):475-84.
25. Papageorghiou, A. T. and Campbell, S. First trimester screening for preeclampsia. Curr Opin Obstet Gynecol. 2006 Dec; 18(6):594-600.
26. Martin, A. M.; Bindra, R.; Curcio, P.; Cicero, S., and Nicolaides, K. H. Screening for pre-eclampsia and fetal growth restriction by uterine artery Doppler at 11-14 weeks of gestation. Ultrasound Obstet Gynecol. 2001 Dec; 18(6):583-6.
27. Gomez, O.; Martinez, J. M.; Figueras, F.; Del Rio, M.; Borobio, V.; Puerto, B.; Coll, O.; Cararach, V., and Vanrell, J. A. Uterine artery Doppler at 11-14 weeks of gestation to screen for hypertensive disorders and associated complications in an unselected population. Ultrasound Obstet Gynecol. 2005 Oct; 26(5):490-4.
28. Poon LC,
29. Poon LC,
30. Poon LC,
31. Perrotin F, Herve P, Wagner N, Descriaud C, Wagner A, Tranquart F. Screening for pre-eclampsia and fetal growth restriction among nulliparous women by uterine artery Doppler at 11-14 weeks' gestation. The value of bilateral notching. Eur J Obstet Gynecol (submitted)
32. Villar, J.; Merialdi, M.; Gulmezoglu, A. M.; Abalos, E.; Carroli, G.; Kulier, R., and de Onis, M. Nutritional interventions during pregnancy for the prevention or treatment of maternal morbidity and preterm delivery: an overview of randomized controlled trials. J Nutr. 2003 May; 133(5 Suppl 2):1606S-1625S.
33. Rumbold, A. R.; Crowther, C. A.; Haslam, R. R.; Dekker, G. A., and Robinson, J. S. Vitamins C and E and the risks of preeclampsia and perinatal complications. N Engl J Med. 2006 Apr 27; 354(17):1796-806.Notes: CORPORATE NAME: ACTS Study Group
34. Poston, L.; Briley, A. L.; Seed, P. T.; Kelly, F. J., and Shennan, A. H. Vitamin C and vitamin E in pregnant women at risk for pre-eclampsia (VIP trial): randomised placebo-controlled trial. Lancet. 2006 Apr 8; 367(9517):1145-54.
35. Duley, L.; Henderson-Smart, D. J.; Meher, S., and King, J. F. Antiplatelet agents for preventing pre-eclampsia and its complications. Cochrane Database Syst Rev. 2007; (2):CD004659.
36. Beaufils, M.; Uzan, S.; Donsimoni, R., and Colau, J. C. Prevention of pre-eclampsia by early antiplatelet therapy. Lancet. 1985 Apr 13; 1(8433):840-2.
37. Ruano, R.; Fontes, R. S., and Zugaib, M. Prevention of preeclampsia with low-dose aspirin -- a systematic review and meta-analysis of the main randomized controlled trials. Clinics. 2005 Oct; 60(5):407-14.
38. Askie, L. M.; Duley, L.; Henderson-Smart, D. J., and Stewart, L. A. Antiplatelet agents for prevention of pre-eclampsia: a meta-analysis of individual patient data. Lancet. 2007 May 26; 369(9575):1791-8.
39. Vainio, M.; Kujansuu, E.; Iso-Mustajarvi, M., and Maenpaa, J. Low dose acetylsalicylic acid in prevention of pregnancy-induced hypertension and intrauterine growth retardation in women with bilateral uterine artery notches. BJOG. 2002 Feb; 109(2):161-7.
40. Subtil, D.; Goeusse, P.; Houfflin-Debarge, V.; Puech, F.; Lequien, P.; Breart, G.; Uzan, S.; Quandalle, F.; Delcourt, Y. M., and Malek, Y. M. Randomised comparison of uterine artery Doppler and aspirin (100 mg) with placebo in nulliparous women: the Essai Regional Aspirine Mere-Enfant study (Part 2). BJOG. 2003; 110:485-91.
41. Rizzo, G.; Capponi, A.; Cavicchioni, O.; Vendola, M., and Arduini, D. First trimester uterine Doppler and three-dimensional ultrasound placental volume calculation in predicting pre-eclampsia. Eur J Obstet Gynecol Reprod Biol. 2007 Oct 2.
42. Plasencia, W.; Maiz, N.; Bonino, S.; Kaihura, C., and Nicolaides, K. H. Uterine artery Doppler at 11 + 0 to 13 + 6 weeks in the prediction of pre-eclampsia. Ultrasound Obstet Gynecol. 2007 Oct; 30(5):742-9.
43. McClure, E. M.; Goldenberg, R. L., and Bann, C. M. Maternal mortality, stillbirth and measures of obstetric care in developing and developed countries. Int J Gynaecol Obstet. 2007 Feb; 96(2):139-46.
44. Fries N, Althuser M, Fontanges M, Talmant C, Jouk PS, Tindel M, Duyme M. Quality control of an image-scoring method for nuchal translucency ultrasonography. Am J Obstet Gynecol 2007; 196: 272. e1-5.
45. Hermida RC, Ayala DE, Calvo C, López JE. Aspirin administered at bedtime, but not on awakening, has an effect on ambulatory blood pressure in hypertensive patients. J Am Coll Cardiol 2005; 46: 975-83.
46. Habli M, Levine RJ, Qian C, Sibai B. Neonatal outcomes in pregnancies with preeclampsia or gestational hypertension and in normotensive pregnancies that delivered at 35, 36, or 37 weeks of gestation. Am J Obstet Gynecol 2007; 197: 406.e1-7.

# Research objectives

## Primary objective

To test the efficacy of low-dose aspirin (160 mg/day), started at an early stage (≤ 15 WA completed), in primiparous patients selected as "at risk" on the presence of bilateral notch and/or high pulsatility index on the uterine arteries, in a pragmatic, double-blind, randomised trial to prevent the occurrence of pre-eclampsia during pregnancy or neonatal hypotrophy ≤ 5^th^ percentile.

## Secondary objectives

- To evaluate the effect of aspirin separately on each of the primary endpoint parameters (pre-eclampsia on the one hand and neonatal hypotrophy on the other hand)
- To evaluate the efficacy of aspirin in preventing early onset and severe forms of pre-eclampsia (occurring before 32 WA)
- To evaluate the efficacy of aspirin in preventing the need for birth before 34 WA, perinatal death (death between the 22^nd^ WA and postnatal day 7)
- To compare in each of the two groups (aspirin and placebo) the delivery route and the frequency of use of loco-regional analgesia.
- To specify the maternal tolerance of the treatment
- To evaluate compliance with the treatment

# Research endpoints

## Primary endpoint

Occurrence of pre-eclampsia during pregnancy or birth weight ≤ 5^th^ percentile:

- Pre-eclampsia, defined as gravid arterial hypertension and proteinuria after 20 WA or in the postpartum period. Arterial hypertension is defined as a systolic blood pressure ≥ 140 mmHg and/or a diastolic pressure ≥ 90 mmHg, measured twice and separated by at least 4 hours. Proteinuria is positive if it is greater than 500 mg/L.
- A birth weight ≤ 5^th^ percentile for the gestational age on AUDIPOG curves adapted to the sex of the newborn and the maternal body mass index (individual curves) [40].

### Secondary endpoints

The following endpoints will be analysed:

- Gestational age at onset of early pre-eclampsia (onset before 32 WA)
- Occurrence of severe pre-eclampsia defined by one of the following criteria:
  - Systolic blood pressure ≥ 160 mmHg and/or diastolic ≥ 110 mmHg
  - Proteinuria ≥ 5 g/day
  - Diuresis ≤ 400 mL/day or ≤ 20 mL/h
  - HELLP syndrome (thrombocytopenia ≤ 100,000 platelets/mm3, AST ≥ 70 IU/L, haemolysis with schizocytes and/or LDH 600 IU/L or haptoglobin < 0.4 g/L)
  - Eclampsia
  - Acute pulmonary oedema
  - RPH (Evocative clinical picture and/or histological confirmation by anatomopathological examination of the placenta)
  - IUFD
- Induced prematurity (triggering and/or maturation, caesarean section)
- Occurrence of perinatal death (death between the 22^nd^ WA and postnatal day 7) not attributable to a cause other than placental insufficiency
- Delivery route: vaginal delivery or caesarean section. Elective caesarean sections (reason for planning) and emergency caesarean sections will be dissociated
- Use of loco-regional analgesia
- Tolerance will be studied from the elements collected from adverse event reports, but also from the "patient" records submitted at the time of inclusion
- Compliance will be evaluated by counting the treatments returned by patients

# Research design

## Description of the research methodology

### Experimental plan

Randomised double-blind parallel-group trial.

The study is planned as a pragmatic trial, in the sense defined by Schwartz and Lellouch (J Chron Dis, 1967;20: 637-648), namely that the women included correspond to the primiparous population at risk (non-inclusion criteria not very restrictive), that the management methods of these women are very flexible (no modification of the usual management, except for the treatment under study) and that the endpoint is medically relevant and simple to collect.

### Description of measures taken to reduce and avoid bias

#### Randomisation

Patients will be randomised to one of the following 2 groups: the low-dose aspirin (160 mg/day) experimental group or the placebo group.

This randomisation will be stratified by investigating centre.

# On a practical level, randomisation will be configured by the Clinical Investigation Centre of the Tours University hospital and will be carried out centrally using the electronic case report form (Capture System software) implemented in this study.

#### Blinding

The study will be conducted in a double-blind manner vs placebo. Thus, neither the patient, nor the investigators, nor the healthcare team will know the nature of the treatment.

An unblinding procedure will be put in place at the level of the electronic case report form.

### Procedure under study

#### Experimental group

Patients in the experimental arm will receive low-dose aspirin (Kardegic® 160 mg/day). The choice of this dose is based on the assumption that the effect of this treatment is most likely to be greatest with the maximum dose that has proven its maternal and foetal safety.

The treatment comes in the form of sachets containing 288 g of DL-lysine acetylsalicylate powder (corresponding to an equivalent of 160 mg of aspirin) to be dissolved in water. The dosage regimen is 1 sachet/day. Treatment will be initiated at enrolment and in any case at a gestational age ≤ 15 WA completed. In the absence of intolerance or major side effects, it will be continued up to 34 WA.

#### Control group

Patients in the control arm will receive a placebo of aspirin that they will take according to the same dosage regimen as that defined for the experimental group.

### Patient follow-up

Apart from treatment (Kardegic® 160 mg/day or placebo), patients will be managed according to the usual methods of pregnancy monitoring [HAS].

## Expected duration of participation of persons and description of the timeline and duration of all test periods, including follow-up, where applicable

The duration of the study is estimated at **53** months (**47** months of inclusion and approximately 6 months of follow-up).

Note that patient follow-up is not fixed, it depends on the inclusion (between 11-15 WA) and the date of delivery.

From the first inclusion, the sponsor must inform the competent authority and the IEC, without delay, of the effective start date of the study (Effective start date = date of signature of the consent by the first patient taking part in the research)

The study end date will be sent by the sponsor to AFSSAPS and the IEC within 90 days. The research end date corresponds to the end of the participation of the last person to take part in the research, or, if applicable, **to the gestational age defined in the protocol.**

# Selection of research subjects

## Inclusion criteria

- Women ≥ 18 years of age
- Primiparous, i.e. never having had a pregnancy ≥ 22 WA.
- Having a singleton pregnancy
- At a gestational age ≤ 15 WA completed i.e. 15^+6^ WA
- Having a bilateral notch ≥ grade II and/or the lowest uterine artery pulsatility index value ≥ 1.7 during the first trimester ultrasound (between 45 and 84 mm CCL)
- Having given their informed consent
- Affiliates or beneficiaries of a social security scheme

## Non-inclusion criteria

- Women wishing to terminate their pregnancy (abortion, with legal gestational age ≤ 14 WA) or for whom termination of pregnancy (therapeutic abortion or premature extraction) is envisaged before the gestational age of 37 WA
- Having a foetal ultrasound abnormality detected during the first trimester ultrasound (foetal malformation or neck ≥ 95^th^ percentile)
- On anticoagulant treatments
- With known allergy or hypersensitivity to Kardegic® or any of its components
- With primary or secondary haemostasis disease responsible for bleeding or at haemorrhagic risk
- With active gastroduodenal ulcer
- With lupus or anti-phospholipid syndrome

## Exclusion criteria

No patient will be excluded from the study, after she has been randomised (intention to treat principle). However, premature termination is possible (see §10.2.1).

## Recruitment procedures

### Selection of recruiting sonographers

The trial is conducted in collaboration with the French College of Foetal Ultrasonography (Collège Français d'Echographie Fœtale - CFEF) which has officially committed (see Appendix). The sonographers involved in patient recruitment will be selected out after validation (in the form of an Evaluation of Professional Practices - EPP). This is a pre-test PPE conducted using a procedure based on a computer tool developed by the CFEF for the quality control of foetal neck measurement [41]. Briefly: each sonographer will be asked to upload to a dedicated website, 20 digital photographs (anonymised for each patient) of Doppler ultrasound measurements of the uterine arteries during the first trimester of pregnancy (10 consecutive photographs with notching and 10 consecutive photographs without notching). Each image must include a B mode image with a colour Doppler mode window of the spectrum measurement site, Doppler window in place, along with the correctly adjusted Doppler spectrum of the uterine artery measured (gain, speed of movement, pulse repetition frequency or PRF) on which the resistance index (RI) and the pulsatility index (PI) are measured.

Each practitioner's images will then be evaluated separately, anonymously, by two experts according to a grid comprising five criteria rated 0, 1 or 2:

- Uterine artery measurement site
- Doppler scroll speed and number of cycles scanned
- PRF adjustment
- Notch assessment
- Index measurement

For an image to be considered satisfactory, it must have been rated at least 6/10.

To be validated, the practitioner must have obtained an average of 13/20, which means that at least 13 of their 20 images must have obtained a score of 6/10. A practitioner who is not validated at the time of a first evaluation may undergo a second subsequent evaluation.

This EPP will allow the practitioner to obtain the "FMC points" necessary for the validation of their regulatory Continuing Medical Training of physicians obligation.

### Recruitment of patients

The sonographers thus validated wishing to participate in the recruitment of patients will undertake to systematically perform, in any primiparous patient, a Doppler ultrasound of the uterine arteries during the first trimester ultrasound. Patients selected in this way will be referred to the nearest investigation centre.

# Course the study

### Patient selection

Sonographers who have validated their EPP will therefore systematically perform a Doppler ultrasound of the uterine arteries in any primiparous patient during the first trimester ultrasound.

In the event of bilateral notching (grade ≥ 2) and/or high pulsatility index (lowest PI value ≥ 1.7), the sonographer will inform the patient of the existence of a trial and will give her:

- a note on the trial,
- the telephone number of the investigation centre to contact,
- a duplicate of the Doppler images of each uterine artery so that the presence of bilateral notching and/or high pulsatility index can be validated by the physician or midwife performing the inclusion visit.

### Inclusion visit

Within each investigation centre, a secretariat (single phone number) will be responsible for responding to patient calls and planning inclusion visits.

At the inclusion visit, the patient will be seen by an investigator: physician or midwife.

After giving the information and answering any questions the patient may still have about the study, the investigator will need to obtain her consent. This document must be dated and signed by the patient and the investigator at this visit before any study-specific evaluation.

Collection of the medical information provided during this consultation (interrogation and standard clinical examination) and by the Doppler images will then serve to validate precisely the study's inclusion and non-inclusion criteria.

Patients having signed a consent and who meet these criteria can thus be included in this study.

Once included, the patient will be randomised to one of the 2 groups using the electronic case report form (see §8.1.2.1). At the end of this randomisation process, a specific prescription with a treatment number (Kardegic® or placebo) will be issued to the patient so that she can collect the entire treatment (161 sachets) for the entire pregnancy from the hospital pharmacy of the centre.

The patient will also be given a card specifying her participation in the study and the contact details necessary for the unblinding request, as well as a "patient" notebook to be returned to the address of the investigation centre after delivery.

### Subsequent follow-up of pregnancy

Subsequent pregnancy follow-up will be carried out according to the usual methods of pregnancy monitoring [HAS]. For a patient followed within the investigation centre itself, the data will be collected from her medical record and the "patient" notebook. For patients not falling into this situation, data will be collected via telephone calls made to the patient and the physician who followed the pregnancy.

## Rules for permanent or temporary discontinuation

### Discontinuation of participation

Patients will be able to withdraw their consent and request to leave the trial at any time and for any reason. In the event of premature discontinuation, the investigator must document the reasons as fully as possible.

The investigator may temporarily or permanently discontinue a patient's participation in the trial for any reason that would be in the best interests of the patient, particularly in the event of serious adverse events.

In the event of a patient lost to follow-up, the investigator will make every effort to resume contact with her and to determine the reasons for the loss of her follow-up.

### Termination of part or all of the research

The study may be terminated prematurely in the event of the occurrence of unexpected serious adverse events requiring a review of the safety profile of the product. Likewise, unforeseen events or new information relating to the product, in view of which the objectives of the study or clinical programme are unlikely to be achieved, may cause the sponsor to prematurely discontinue the study.

The Tours Regional University Hospital reserves the right to interrupt the study at any time if it turns out that the inclusion objectives have not been achieved.

In the event of premature termination of the study, the information will be sent by the sponsor within 15 days to AFSSAPS and the IEC.

### Follow-up modalities for a patient leaving the study

The withdrawal of a patient from the study will not change the usual methods of pregnancy monitoring [HAS]. In the event of an adverse event, whether serious or not, precise follow-up may be considered depending on the seriousness of the adverse event and the severity. The Supervisory Committee will specify the modalities for follow-up on a case-by-case basis.

# Treatments given to patients taking part in the research

## Description of the treatment(s) required to carry out the research

### Experimental medicinal product(s)

#### Identification of treatments

Women in the experimental arm will receive sachets of Kardegic® 160 mg/day. The treatment comes in the form of sachets containing 288 g of DL-lysine acetylsalicylate powder (corresponding to an equivalent of 160 mg of aspirin) to be dissolved in water.

The women in the control arm will receive sachets of an aspirin placebo (see technical offer from Bertin Pharma), which they will take according to the same dosage regimen as that defined for the experimental group.

#### Packaging and labelling

The treatments will be packaged in the form of a kit of 161 sachets.

The treatments will be packaged in order to conform to blinding and they will be labelled in accordance with Article 7 of the decree of 24 May 2006 laying down the content of the labelling of investigational medicinal products.

#### Manufacturing and distribution of treatments

Placebo manufacture and packaging along with that of Kardegic will be carried out by the contractor BERTIN Pharma.

The Internal Pharmacy (IP) of the Bretonneau Hospital of the Tours University hospital will coordinate the IPs for the recruiting centres and in particular their supply.

Each IP will then be responsible for the treatment of the patients included in their centre: delivery, management and accounting of returns, destruction and request for resupply.

#### Administration

The dosage regimen is 1 sachet/day. Treatment will be initiated at enrolment and in any case at a gestational age ≤ 15 WA completed. In the absence of intolerance or major side effects, it will be continued up to 34 WA.

#### Dosage regimen adaptation

Not applicable

#### Precautions for use

The precautions for use are described in the Kardegic® Summary of Product Characteristics (SPC).

Patients will also be advised to take this treatment in the evening during their meal [42].

## Medicinal products and treatment authorised and prohibited under the protocol

Since this is a pragmatic trial, the concomitant treatments authorised are the usual treatments.

For most teams, performing amniocentesis does not require the interruption of low-dose aspirin therapy. For this reason, there will be no unblinding for this type of procedure if an indication is made during treatment. However, it is recommended, as a precautionary measure, in this case, to avoid transplacental passage of the amniocentesis needle.

## Method of monitoring compliance with treatment

Compliance will be evaluated from the accounting of the treatments provided. For this, patients will be asked to return their treatment boxes at the time of childbirth or during a monitoring consultation after discontinuation of treatment (after 34 WA).

## Storage conditions for investigational medicinal products

### Description of storage

Each of the IPs will store the medicinal products in a specific "Clinical Trials" room, separate from the storage of the other medicinal products. Medicines will be stored in a dry place, at a temperature below 25°C and their expiry date will be observed.

## Stock management, replenishment

The stocks will be managed by the IP of the Bretonneau Hospital of the Tours University hospital. An initial allocation will be set up in the IPs of the recruiting centres.

Each of the pharmacies will be replenished according to the inclusions made by each of the centres.

# Statistical considerations

## Number of patients to include

The population size is calculated as follows:

- pre-eclampsia occurs in 5-7% of primiparous women [19]
- the presence of bilateral notching and/or a high pulsatility index is predictive of the occurrence of pre-eclampsia, and this with a sensitivity of 75% and a specificity of 67% [28]

Hence, based on these data, out of every 100 primiparous women, the following numbers are expected to occur:

|  | Pre-eclampsia | No pre-eclampsia |  |
| --- | --- | --- | --- |
| Bilateral notching and/or high PI | 4.5 | 31 | 35.5 |
| No bilateral notching | 1.5 | 63 | 64.5 |
|  | 6 | 94 | 100 |

Thus, out of every 100 primiparous women, 35.5 women are expected to present with bilateral notching and/or a high pulsatility index, of which 4.5 will go on to develop pre-eclampsia.

The expected rate of pre-eclampsia in the control group is therefore 4.5/35.5 or 12.7%.

- Hypotrophy, defined by a birth weight ≤ 5^th^ percentile, occurs more frequently in primiparous than in multiparous women [40]. Thus, although intrinsically (probabilistic definition of the disease), the prevalence of hypotrophy as defined is 5%, in primiparous women a rate of hypotrophy of approximately 7% is expected [43].
- the presence of bilateral notching and/or a high pulsatility index is predictive of the occurrence of hypotrophy, and this again with a sensitivity of 55% and a specificity of 67% [28].

Hence, based on these data, out of every 100 primiparous women, the following numbers are expected to occur:

|  | Hypotrophy | No hypotrophy |  |
| --- | --- | --- | --- |
| Bilateral notching and/or high PI | 3.85 | 31 | 34.85 |
| No bilateral notching | 3.15 | 63 | 66.15 |
|  | 7 | 94 | 100 |

Hence, out of every 100 primiparous women, 34.85 women with bilateral notching and/or a high pulsatility index are expected, of which 3.85 will give birth to a hypotrophic child.

The expected rate of hypotrophy in the control group is therefore 3.85/34.85 or 11.0%. Moreover, it is expected that for 20% of women developing pre-eclampsia we will also observe hypotrophy.

Thus, in the control group, we expect to observe [12.7 + (11.0-0.2*12.7)] or 21.2% of events (i.e. pre-eclampsia or hypotrophy).

Our hypothesis is that aspirin will reduce the occurrence of events by 15% [32]. The expected difference is therefore 3.2%, i.e. 21.2% of events are expected in the control group compared to 18.0% in the experimental group.

Considering the risks of type 1 and 2 errors set at 5 and 20% respectively, we must include 2415 women per group (nQuery Advisor® 6.0).

Additionally, this study plans to carry out intermediate analyses. The hypotheses relating to the control group are poorly documented, which led us to rule out a triangular test experimental design. Indeed, we know that poor advance knowledge of the success rate in the control group is potentially deleterious for the study (Sebille *et al*, Impact of a mis-specification of the response rate under standard treatment in sequential clinical trials. [*Fundam Clin Pharmacol*.](javascript:AL_get(this, 'jour', 'Fundam Clin Pharmacol.');) 2005;19:569-578). Finally, we decided to plan this study according to a Peto scheme (Peto R, Pike MC, Armitage P. Design and analysis of randomized clinical trials requiring prolonged observation of each patient. Br J cancer 1976;34:585-612. PMID).

Four intermediate analyses are planned, such that if the trial is discontinued after one of these intermediate analyses, the success rates associated with each of the groups are still accurately estimated. These intermediate analyses will be carried out every 1000 inclusions.

Carrying out an intermediate analysis leads to an increase in the total number of subjects to be included (if the study goes to its conclusion), such that the overall type I error (i.e. taking into account all the analyses) remains at the nominal value. Considering alpha at 5% and beta at 20%, the inflation parameter is 1.024 (Jennison *et al* (2000). Group sequential methods with applications to clinical trials. Chapman & Hall/CRC: Boca Raton), which leads us to include 2415*1.024 or 2473 patients per arm.

Finally, it is expected that for 0.5% [40] of women, the pregnancy will not be carried to term (medical termination of pregnancy). These women will not be taken into account in the primary analysis. We must therefore increase the number such that at the end of the study, we have 2473 analysable women per arm. This therefore leads us to include 2473/0.995 or 2486 women per arm.

Ultimately, 4972 women are expected to be included in this trial.

## Data analysis: general

Statistical analysis will be carried out according to a pre-established analysis plan.

The analysis will be conducted by considering the eligible patients, i.e. any woman for whom the pregnancy has not been carried to term (medical termination of pregnancy, IMG) will be excluded from the analysis. These exclusions will not introduce a bias because we can rule out the hypothesis that malformations inducing an abortion are due to aspirin (see 2.6.4). We thus expect to have the same proportion of therapeutic abortions in both groups as these are independent of the investigational medicinal product, enabling us to exclude these patients without risk of bias (Fergusson *et al*. Post-randomisation exclusions: the intention to treat principle and excluding patients from analysis. *Bmj* 2002;325:652-654).

As the population is of pregnant women, the risk of loss to follow-up is low or non-existent. Should some patients be lost to follow-up, however, they would be taken into account in the analysis according to the strategy of maximum bias, namely that they would be considered in failure in the experimental arm and in failure in the control arm.

Intermediate analyses will be conducted every 1000 inclusions. We plan to conduct 4 intermediate analyses, whose results will be presented to the independent supervisory committee of the study.

A per-protocol analysis will also be performed as a secondary analysis. The women to be excluded from this per-protocol analysis will be identified during a blind review.

A statistical analysis report will be prepared, incorporating all the elements that must be reported, as recommended by the CONSORT Statement (<http://www.consort-statement.org/> - Accessed on 24.10.11).

## Description of samples at inclusion

The randomisation groups will be compared using descriptive statistics. No statistical tests will be performed.

## Primary endpoint analysis

Rates of pre-eclampsia during pregnancy or birth weight ≤ 5^th^ percentile will be compared using a chi-square test. An adjustment for stratification factors will also be made as part of a logistic regression model.

Intermediate analyses (one every 1000 inclusions) will be performed at the thresholds of 0.001 and 0.049 for the last one (Peto rule).

The final analysis (if it needs to be carried out) will be conducted at the end of the 4972 inclusions. It will be performed at the 0.049 threshold, which means that a significant difference will be concluded only if the degree of significance is less than 0.049.

Finally, because intermediate analyses will be carried out, it will be necessary to adjust the estimates of the effect, the confidence interval of this effect, and the degree of significance. This will be achieved through implementation of the Lan & De Mets method (Discrete sequential boundaries for clinical trials. *Biometrika*. 1983;70:659-663).

## Secondary analyses

The various components of the primary endpoint will be compared, in accordance with the scientific guidelines for the analysis of "composite endpoints" (Freemantle *et al*. JAMA 2003;289:2554-2559).

We will thus compare the two groups in terms of the occurrence of:

- Pre-eclampsia
- Hypotrophy

Moreover, subgroup analyses will be performed considering gestational age and body mass index (BMI).

A secondary analysis will also be conducted distinguishing two other components of the primary endpoint, namely early and severe pre-eclampsia (placental and maternal).

Rates of caesarean section and use of locoregional analgesia will be compared using chi-square tests.

Compliance will be described in each group.

Regarding tolerance, the analysis will be mainly descriptive, with possible chi-square tests or exact Fisher tests.

## Person responsible for the analysis

The analysis will be carried out under the responsibility of Bruno Giraudeau, INSERM CIC 202. The data will be analysed in SAS, version 9.2 or later.

# Safety evaluation

## Procedures in place for recording and reporting adverse events

### Definitions

- **Adverse event (AE)**: any harmful manifestation occurring in a person taking part in biomedical research, whether or not this manifestation is related to the research or to the investigational drug(s) to which this research relates.
- **Serious Adverse Event (SAE)**: severity is defined by one of the following findings:
  - death
  - threat to life (immediate threat to life at the time of the event, regardless of the consequences of corrective or palliative therapy)
  - significant or lasting impairment or disability
  - hospitalisation
  - extension of hospitalisation
  - congenital malformation/anomaly
  - potentially serious event (adverse clinical event or laboratory result of a serious nature or considered as such by the investigator)

Certain circumstances requiring hospitalisation do not fall under the severity criterion "hospitalisation / extension of hospitalisation" and should not be declared as SAEs:

- - hospitalisation predefined by the protocol,
  - admission for social or administrative reasons,
  - visit to day hospital,
  - hospitalisation for routine treatment or monitoring of the studied disease not associated with a deterioration of the patient's condition,
  - hospitalisation for medical or surgical treatment scheduled before the start of the research.
- **Adverse reaction (AR)**: any harmful and unwanted reaction to an investigational medicinal product, regardless of the dose administered.
- **Serious adverse reaction (SAR)**: serious adverse event attributable to an investigational medicinal product.
- **Unexpected adverse reaction**: an adverse reaction whose nature, severity, intensity or course does not agree with the information contained in the summary of product characteristics for an authorised investigational medicinal product or, in the case of an unauthorised medicinal product, in the investigator's brochure.
- **Imputability**: relationship between the AE and the study treatment. An AE related to the investigational medicinal product will become an AR. Factors to consider when determining imputability include:
  - chronology of events,
  - the disappearance of the AE when stopping the medicinal product(s) and/or its reappearance in the event of re-administration,
  - the pharmacodynamics and pharmacokinetics of the medicinal products,
  - the notion of a history of a similar events when administering the medicinal product or a medicinal product of the same class,
  - existence of another aetiology.

**- New development: Imputability:** any safety data that could significantly change the evaluation of the benefit-risk ratio of an investigational medicinal product or of the trial, or which could lead to the consideration of changes in the administration of the medicinal product or the conduct of the trial, such as:

- any clinically significant increase in the frequency of occurrence of an expected serious adverse reaction;
- SUSARs in participants who have completed the trial and are reported by the investigator to the sponsor, as well as any follow-up reports;
- any new developments concerning the course of the clinical trial or the development of the investigational medicinal product, where such new developments are likely to affect the safety of the subjects.

### Investigator's responsibility

#### Notification of serious adverse events

##### Information to be sent to the sponsor

Each SAE will be described on the form provided for this purpose, trying to be as exhaustive as possible. The following information will be submitted:

- patient identification (number, code, date of birth, date of inclusion, sex, weight, height),
- severity of the SAE,
- SAE start and end dates,
- clear and detailed description of the SAE (diagnosis, symptoms, intensity, chronology, actions taken and results),
- outcome of the SAE,
- ongoing diseases or relevant history of the patient,
- treatments received by the patient,
- causal relationship between the SAE and the investigational medicinal product(s), comparator(s), possible associated treatments, research or other criteria.

Whenever possible, the investigator must also attach the following to the SAE report:

- a copy of the hospitalisation or extension of hospitalisation report,
- where applicable, a copy of the autopsy report,
- a copy of all the results of additional examinations carried out, including the relevant negative results, with the normal laboratory values,
- any other documents deemed useful and relevant.

These documents will be anonymised and will bear the patient identification number.

Each adverse event will be monitored until complete resolution (stabilisation at a level deemed acceptable by the investigator or return to the previous state) even if the subject has left the trial.

##### Protocol specifics

Expected SAEs (related to the patient's condition) will not require (in agreement with the health authorities) an immediate declaration. The SAEs concerned are hospitalisations for the following reasons:

- pre-eclampsia
- severe pre-eclampsia defined by one of the following criteria:
- Systolic blood pressure ≥ 160 mmHg and/or diastolic ≥ 110 mmHg
- Proteinuria ≥ 5 g/day
- Diuresis ≤ 400 mL/day or ≤ 20 mL/h
- Eclampsia
- HELLP syndrome (thrombocytopenia ≤ 100,000 platelets/mm3, AST ≥ 70 IU/L, haemolysis with schizocytes and/or LDH 600 IU/L or haptoglobin < 0.4 g/L)
- Acute pulmonary oedema
- RPH
- IUGR
- Caesarean section

##### Sponsor notification requirements

Any SAE, regardless of its causal relationship with the treatment(s) of the trial or research (with the exception of those identified in the protocol as not requiring immediate reporting), must be declared to the Clinical Investigation Centre of the Tours University hospital by fax +33 (0)2.47.47.46.62.

A Vigilant (Hédia BOIVIN or Annie-Pierre JONVILLE-BERA) can be reached by phone at +33 (0)2.47.47.70.98 or +33 (0)2.47.47.36.01.

##### Sponsor notification deadline

The investigator shall notify the sponsor, without delay from the day on which they becomes aware of them, of all serious adverse events occurring in the trial, with the exception of those identified in the protocol as not requiring immediate reporting.

The initial declaration may be followed by additional relevant information within **8 days** in the event of a fatal or life-threatening event and within **15 days** in other cases.

##### Sponsor notification period

The investigator is responsible for noting and reporting all SAEs occurring during the entire study: from the date of signing of the consent and for the entire duration of the participant's follow-up planned by the trial.

Furthermore, regardless of the time frame after the end of the study, any SAE potentially due to the research must be reported to the sponsor if no cause other than the research can reasonably be attributed to it (e.g. serious effects that may occur a long time after exposure to the medicinal product, such as cancers or congenital anomalies).

#### Notification of non-serious adverse events

All other AEs will be reported on the "adverse event" form in the Case Report Form.

### Sponsor's responsibility

#### Analysis of serious adverse events

The sponsor must evaluate:

- SAE causality (all adverse events for which the investigator or sponsor considers that a causal relationship with the investigational medicinal product(s) can be reasonably envisaged, are considered to be suspected adverse reactions. If the sponsor and investigator reach different evaluations, the two opinions are mentioned on the declaration sent to the competent authority if this declaration is necessary),
- and their expected or unexpected nature using the reference document (investigator's brochure or SPC) in force.

#### Imputability rating

In accordance with ICH guidelines on the management of adverse events in clinical studies - ICH E2B(R3), version of 12 May 2005 - an evaluation of imputability is carried out for any declared SAE. The following rating method is used:

- **Unrelated**: the event appears within a time frame incompatible with the administration of the medicinal product and/or there is a sufficient amount of information showing that the observed reaction is unrelated to the medicinal product and/or there is a plausible alternative explanation.
- **Questionable relationship**: the event has a chronology (onset, course) that is not compatible with the administration of the medicinal product and is likely due to factors other than the medicinal product, such as the patient's clinical condition or the concomitant administration of other medicinal products.
- **Possible relationship**: the event appears within a compatible time frame after administration of the medicinal product and, although this latter's responsibility cannot be ruled out, other factors may be involved, such as the patient's clinical condition or the concomitant administration of other medicinal products. Information concerning progression upon discontinuation of the medicinal product may be missing or inconclusive.
- **Probable relationship**: the event appears within a compatible time frame after administration of the medicinal product. It cannot reasonably be attributed to another factor, such as the patient's clinical condition or concomitant medication. Progression following discontinuation must be clinically compatible. Information on the reintroduction of the medicinal product is not essential.
- **Highly probable relationship**: the event appears within a highly suggestive time frame after administration of the medicinal product. It cannot be explained by any other factor, such as the patient's clinical condition or concomitant medication. Progression following discontinuation must be clinically compatible. The event can be explained pharmacologically or pathophysiologically, or has recurred at re-administration of the medicinal product.

Adverse events with a questionable, possible, probable or highly probable relationship with the investigational medicinal product(s) are considered to be related to the investigational medicinal product(s). If they are unexpected, they are qualified as SUSARs and must be declared by the sponsor (see next paragraph).

#### Declaration of suspected unexpected serious adverse reactions

The sponsor reports all suspected unexpected serious adverse reactions (SUSARs) to Eudravigilance (European pharmacovigilance database), the French Health Authorities (AFSSAPS), the independent ethics committee (IEC) and the investigators.

Regulatory declaration is made within a maximum period of:

- **7 calendar days** for suspected unexpected fatal or life-threatening serious adverse reactions. In this case, relevant additional information must be sought and transmitted within a further **8 days**.

- **15 calendar days** for all other suspected unexpected serious adverse reactions. Likewise, relevant additional information must be sought and submitted within a further **8 days**.

Detailed records of all adverse events reported by investigators may be transmitted to AFSSAPS at its request.

#### Transmission of annual safety reports

On the anniversary date of the trial authorisation issued by the Health Authorities, the sponsor drafts a safety report including:

- a list of serious adverse reactions likely to be related to the investigational medicinal product(s) in the trial, including suspected unexpected and expected serious adverse reactions,

- a concise and critical analysis of the safety of the participants taking part in the research.

This report may be submitted to the coordinating investigator for approval. It is sent to the competent authorities (AFSSAPS) and the IEC within **60 days** of the anniversary date of the trial authorisation.

#### Transmission of semi-annual safety reports

Every six months, the sponsor sends to the IEC, with a copy to AFSSAPS, a list of the SUSARs that have occurred in the research outside the national territory, along with the SUSARs that have occurred in another research on the same investigational medicinal product that it is sponsoring.

#### Declaration of other safety-related data

The promoter must declare any safety data or new facts to AFSSAPS and the IEC concerned as soon as possible and at the latest within **15 calendar days** from the time the sponsor became aware. Additional relevant information must be submitted within a further **8 days.**

### Supervisory Committee

The Independent Supervisory Committee (ISC) is an advisory committee responsible for advising the sponsor and the coordinator or principal investigator of the study on the safety of a clinical trial.

This committee analyses the study data, issues an opinion on the benefit/risk ratio and proposes the following corresponding measures:

- continuation of the protocol,
- need for additional analyses (statistics, SAEs to document, etc.),
- need for amendment (major changes to the protocol in terms of safety or data analysis, or safety developments)
- discontinuation of the study

The opinion of the ISC is sent to the sponsor, the study coordinator, the Independent Ethics Committee and AFSSAPS. Upon receipt of the opinion of the ISC, the Sponsor deliberates and makes its decision. It is customary for the Sponsor to conform to the position of the ISC, but it is not obliged to do so because this position is advisory.

## Modalities and duration of patient follow-up after the occurrence of adverse events

Each adverse event will be monitored until complete resolution (stabilisation at a level deemed acceptable by the investigator or return to the previous state) even if the subject has left the trial.

# Right of access to source data and documents

## Data access

In accordance with GCP:

- the sponsor is responsible for obtaining the agreement of all parties involved in the research to guarantee direct access to all places where research is conducted, to source data, to source documents and reports for the purposes of quality control and audit by the sponsor,

- the investigators will make available to the persons responsible for the monitoring, quality control or audit of biomedical research, the documents and individual data strictly necessary for this control, in accordance with the laws and regulations in force (Articles L.1121-3 and R.5121-13 of the Public Health Code).

## Source documents

The source documents, being defined as any original document or object making it possible to prove the existence or accuracy of a data or fact recorded during the [clinical study](http://134.157.220.13/urcest/sous_cadre.php?fich=Lexique/E.htm#essaiclinique), will be kept for 15 years by the investigator or by the hospital in the case of a hospital medical record.

## Data confidentiality

In accordance with the provisions concerning the confidentiality of data to which persons responsible for quality control of biomedical research have access (Article L.1121-3 of the Public Health Code), in accordance with the provisions relating to the confidentiality of information concerning in particular the nature of the investigational medicinal products, the trials, the patients taking part in them and the results obtained (Article R. 5121-13 of the Public Health Code), persons with direct access will take all the necessary precautions to ensure the confidentiality of information relating to the investigational medicinal products, the trials, the patients taking part in them and in particular as regards their identity and the results obtained.

These people, along with the investigators themselves, are subject to professional secrecy (under the conditions defined by Articles 226-13 and 226-14 of the Criminal Code).

During or after the biomedical research, the data collected on the individuals taking part and passed on to the sponsor by the investigators (or any other specialized stakeholders) will be rendered anonymous. They must under no circumstances include the full names of the persons concerned or their addresses. Only the first letter of the subject's first and last name will be recorded, along with a unique encrypted number for the study indicating the order of inclusion of subjects.

The sponsor will ensure that each patient taking part in the research has agreed in writing to allow access to their personal data that are strictly necessary for the quality control of the research.

# Quality assurance and control

A Clinical Research Associate (CRA) appointed by the sponsor will ensure the proper conduct of the study, the collection of data generated in writing, their documentation, recording and reporting, in accordance with the Standard Operating Procedures implemented within the Clinical Investigation Centre of the Tours University hospital and in accordance both with Good Clinical Practice and with the laws and regulations in force.

The investigator and the members of their team agree to make themselves available during the Quality Control visits carried out at regular intervals by the Clinical Research Associate. During these visits, the following elements will be reviewed:

- informed consent
- compliance with the study protocol and the procedures defined therein
- quality of the data collected in the Case Report Form: completeness, accuracy, missing data, consistency of the data with the source documents (medical records, appointment books, originals of laboratory results, etc.)
- management of any products.

Furthermore, the investigators undertake to accept the quality assurance audits carried out by the sponsor, along with the inspections carried out by the Competent Authorities. All data, documents and reports may be subject to regulatory audits and inspections, notwithstanding any objections based on medical confidentiality.

# Ethical considerations

## Independent Ethics Committee

The protocol, the information form and the study consent certificate will be submitted for opinion to the Independent Ethics Committee – Ouest 1 (Centre Region).

The notification of the favourable ruling by the IEC will be forwarded to the study sponsor and the Competent Authority. A request for authorisation will be sent by the Sponsor to AFSSAPS before the start of the study.

## Substantive changes

Any substantive changes made to the protocol by the investigator must be approved by the sponsor. This latter must obtain, prior to implementation, a favourable ruling from the IEC and an authorisation from AFSSAPS within the framework of their respective competences. A new consent from the patients participating in the research will be collected if necessary.

## Patient information and written informed consent form

The patients will be informed in a complete and fair manner, in understandable terms, of the objectives and constraints of the study, the possible risks involved, the necessary surveillance and safety measures, their rights to refuse to participate in the study and the possibility of withdrawing at any time.

All this information is contained in an information and consent form given to the patient. The patient's free, informed and written consent will be obtained by the investigator, physician or midwife prior to final inclusion in the study. A copy of the information and consent form signed by both parties will be issued to the patient, the investigator will keep the original. At the end of the study, a copy will be placed in a sealed tamper-proof envelope containing all consent forms, which will be archived by the sponsor.

## Definition of the exclusion period

There are no plans to exclude patients from participation in another clinical research other than a study on placental insufficiency. The exclusion period for studies of placental insufficiency ends within 72 hours of delivery.

## Care related to the research

There is no change in the care of women included in the study, who will therefore be monitored at the same rate as are usually pregnant women.

## Patient compensation

None

## Enrolment in the national biomedical research patient registry

Not applicable

# Data processing and storage of documents and data

## Case report form

All information required by the protocol must be recorded in the case report forms. The data should be collected as they are obtained, and recorded in these case report forms explicitly. Any missing data must be coded.

This electronic case report form will be implemented in each of the centres through an Internet-based data collection medium. The investigators will be issued with a help document for the use of this tool.

Completion of the case report form via the internet by the investigator thus allows the CRA to view the data quickly and remotely. The investigator is responsible for the accuracy, quality and relevance of all data entered. Moreover, when they are entered, these data are immediately verified through consistency checks. As such, the investigator must validate any change in value in the CRF. These changes are tracked by an audit trail. A justification may be optionally included in the comments. A paper printout will be requested at the end of the study, authenticated (dated and signed) by the investigator. A copy of the authenticated document intended for the sponsor must be archived by the investigator.

## Data input and processing

The data are entered directly at the investigation centres via the electronic case report form.

## CNIL

As a result of the telephone follow-up carried out and, consequently, of the collection of personal data (surname, first name, telephone number) that this implies, a request for an opinion will be submitted to CCTIRS and CNIL.

## Archiving

Pursuant to Article R. 1123-61 of the Public Health Code, the sponsor and the centres participating in the study will keep the documents and data relating to the research that are specific to them (see *Decision of 24 November 2006 laying down the rules of good clinical practice for biomedical research relating to medicinal products for human use*) for at least fifteen years after the end of the biomedical research or its early termination without prejudice to the laws and regulations in force.

In each of the centres, these documents will be kept in the principal investigator's department until the end of the practical usefulness period. At the end of this period, all the documents to be archived may be transferred to the central archives of each of the centres, in accordance with institutional practices.

For the sponsor, these documents will also be kept until the end of the practical usefulness period. At the end of this period, all the documents to be archived, as defined in the procedure for "filing and archiving documents related to biomedical research" of the TOURS University hospital, will be transferred to the archiving site (Central Archives Service – Trousseau Hospital) and will be placed under the responsibility of the Sponsor for 15 years after the end of the study.

They must not be moved or destroyed without the sponsor's consent. At the end of these 15 years, the sponsor will be consulted for destruction. All data, documents and reports may be subject to audit or inspection.

# Financing and insurance

## Study budget

The funding allocated to this study is €918k. The financial management of these credits will be placed under the responsibility of the Directorate of Medical Affairs and Research of the Tours University Hospital.

## Insurance

For the duration of the study, the Sponsor will take out insurance guaranteeing its own civil liability as well as that of any physician involved in carrying out the trial. It will also ensure full compensation for the harmful consequences of the research for the patient participating in it and their beneficiaries, unless it can prove that the damage is not attributable to its fault or to that of any contributor, notwithstanding any objections involving the actions of a third party or the voluntary withdrawal of the patient who had initially consented to participate in the research.

# Study feasibility

The feasibility of this study lies in the following:

- Eligible women will be identified by the sonographers who have validated the CFEF EPP, which is associated with and a stakeholder in the study (see commitment appended). The choice of centres was made because of the close relationship between these private practice sonographers and hospital obstetricians, thus ensuring an operational recruitment process.
- The trial was designed as a pragmatic trial. The study procedure is limited to the administration of aspirin only. No changes are made to the usual follow-up of these pregnant women.
- The data collected will be limited in number, the collection will be done via an electronic case report form and at each centre, CRT time is budgeted to ensure the management of the inclusion visit (in association with the investigating physician(s)) and the collection of data during or after the delivery.
- The women who will be included in this study are pregnant women and are in fact a "captive" population. As such, there will therefore be no procedure to be implemented to ensure follow-up of patients and to limit the number of lost to follow-up.

In terms of numbers, the annual inclusion forecasts are as follows: these forecasts only refer to patients giving birth at the investigation centre. However, the decision to open the inclusion to all patients regardless of their place of delivery, means that the figures announced are probably somewhat underestimated compared to the real possibilities of inclusion.

| Centre | Annual number of 1^st^ trimester ultrasounds or childbirths | Number of primiparous women  (a) | Number of primiparous women with bilateral notching or high PI  (b) | Number of women likely to be included (c) |
| --- | --- | --- | --- | --- |
| Necker (Primafacie) | 5000 | 2250 | 788 | 260 |
| Centre Européen de diagnostic et d’Exploration de la femme (78) | 5000 | 2250 | 788 | 260 |
| Lille | 5000 | 2250 | 788 | 260 |
| Bordeaux | 4500 | 2025 | 709 | 234 |
| Tours | 4000 | 1800 | 630 | 208 |
| Toulouse | 4000 | 1800 | 630 | 208 |
| Orléans | 4000 | 1800 | 630 | 208 |
| Nantes | 4000 | 1800 | 630 | 208 |
| Lyon | 3000 | 1350 | 473 | 156 |
| Montpellier | 3000 | 1350 | 473 | 156 |
| Caen | 3000 | 1350 | 473 | 156 |
| Clermont-Ferrand | 2500 | 1125 | 394 | 130 |
| Nimes | 2000 | 900 | 315 | 104 |
| Cabinet Mosaique Santé (Blois) | 1500 | 675 | 236 | 78 |

1. 45% of women who give birth are primiparous
2. 35% of primiparous women have bilateral notching and/or high PI
3. The sonographers will only recruit some of the women who give birth at the recruiting centres; some women will refuse to take part in the study. Ultimately, the assumption is that one third of the women eligible for the study will actually be included. However, the choice not to limit inclusion to patients giving birth in the study centre means that the estimation of the latter parameter is the low hypothesis.

The total number of women likely to be included is a little over 2,500 patients per year. A recruitment period of 2 ½ years is therefore planned in order to ensure the recruitment of the 4972 patients, constituting the number planned for the study.

# Rules of publication

Communications and scientific reports for this study will be carried out under the supervision of the coordinating investigator of the study with the consent of the responsible investigators. The co-authors of the report and publications will be the investigators and clinicians involved, in proportion to their contribution to the study, along with the biostatistician and research associates.

The CFEF will be clearly mentioned in any publications resulting from this trial.

Publication rules follow international guidelines (N Engl J Med 1997; 336: 309-315). The study will be registered on an open access website (Clinical trial) before the inclusion of the first patient in this study.

# Appendices


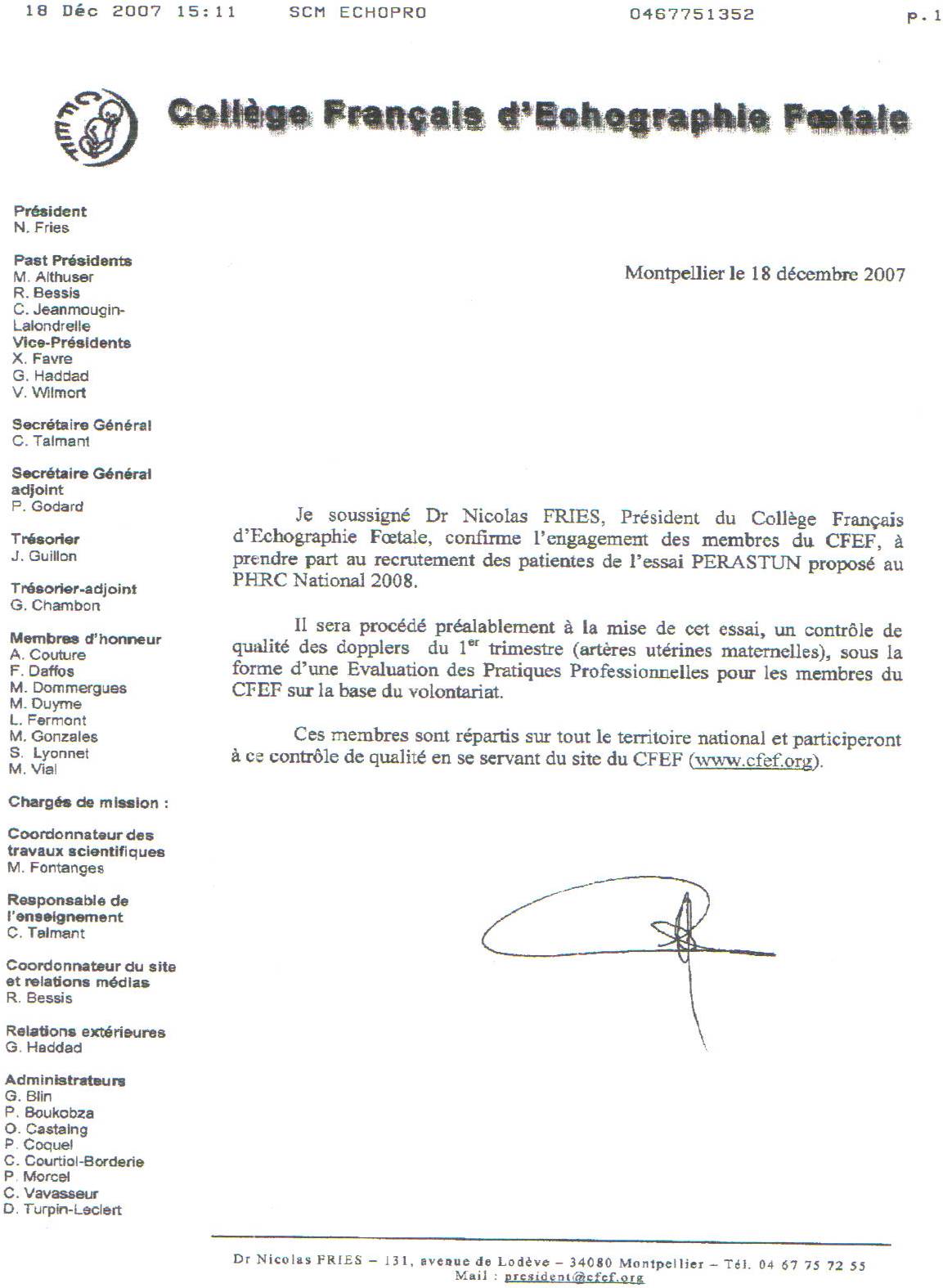

Supplement: S3 File — (DOC) [file pone.0275129.s006.doc]
